# Causes and incidence of community-acquired serious infections among young children in south Asia (ANISA): an observational cohort study

**DOI:** 10.1016/S0140-6736(18)31127-9

**Published:** 2018-07-14

**Authors:** Samir K Saha, Stephanie J Schrag, Shams El Arifeen, Luke C Mullany, Mohammad Shahidul Islam, Nong Shang, Shamim A Qazi, Anita K M Zaidi, Zulfiqar A Bhutta, Anuradha Bose, Pinaki Panigrahi, Sajid B Soofi, Nicholas E Connor, Dipak K Mitra, Rita Isaac, Jonas M Winchell, Melissa L Arvay, Maksuda Islam, Yasir Shafiq, Imran Nisar, Benazir Baloch, Furqan Kabir, Murtaza Ali, Maureen H Diaz, Radhanath Satpathy, Pritish Nanda, Bijaya K Padhi, Sailajanandan Parida, Aneeta Hotwani, M Hasanuzzaman, Sheraz Ahmed, Mohammad Belal Hossain, Shabina Ariff, Imran Ahmed, Syed Mamun Ibne Moin, Arif Mahmud, Jessica L Waller, Iftekhar Rafiqullah, Mohammad A Quaiyum, Nazma Begum, Veeraraghavan Balaji, Jasmin Halen, A S M Nawshad Uddin Ahmed, Martin W Weber, Davidson H Hamer, Patricia L Hibberd, Qazi Sadeq-ur Rahman, Venkat Raghava Mogan, Tanvir Hossain, Lesley McGee, Shalini Anandan, Anran Liu, Kalpana Panigrahi, Asha Mary Abraham, Abdullah H Baqui

**Affiliations:** a Department of Microbiology, Child Health Research Foundation, Dhaka Shishu Hospital, Sher-E-Bangla Nagar, Dhaka, Bangladesh; b Centers for Disease Control and Prevention, Respiratory Diseases Branch, Atlanta, GA, USA; c Maternal and Child Health Division, icddr,b, Dhaka, Bangladesh; d Johns Hopkins Bloomberg, School of Public Health, Johns Hopkins University, Baltimore, MD, USA; e Department of Child and Adolescent Health and Development, World Health Organization, Geneva, Switzerland; f Department of Paediatrics and Child Health, Aga Khan University, Karachi, Pakistan; g Christian Medical College, Bagayam, Vellore, India; h Center for Global Health and Development, College of Public Health, University of Nebraska Medical Center, Omaha, NE, USA; i Asian Institute of Public Health, Bhubaneswar, India; j Ramachandra Bhanj Medical College, Manglabag, Cuttack, Odisha, India; k Child and Adolescent Health and Development Division, World Health Organization Regional Office for Europe, Copenhagen, Denmark; l Department of Global Health and Center for Global Health and Development, Boston University School of Public Health, Boston, MA, USA

## Abstract

**Background:**

More than 500 000 neonatal deaths per year result from possible serious bacterial infections (pSBIs), but the causes are largely unknown. We investigated the incidence of community-acquired infections caused by specific organisms among neonates in south Asia.

**Methods:**

From 2011 to 2014, we identified babies through population-based pregnancy surveillance at five sites in Bangladesh, India, and Pakistan. Babies were visited at home by community health workers up to ten times from age 0 to 59 days. Illness meeting the WHO definition of pSBI and randomly selected healthy babies were referred to study physicians. The primary objective was to estimate proportions of specific infectious causes by blood culture and Custom TaqMan Array Cards molecular assay (Thermo Fisher, Bartlesville, OK, USA) of blood and respiratory samples.

**Findings:**

6022 pSBI episodes were identified among 63 114 babies (95·4 per 1000 livebirths). Causes were attributed in 28% of episodes (16% bacterial and 12% viral). Mean incidence of bacterial infections was 13·2 (95% credible interval [CrI] 11·2–15·6) per 1000 livebirths and of viral infections was 10·1 (9·4–11·6) per 1000 livebirths. The leading pathogen was respiratory syncytial virus (5·4, 95% CrI 4·8–6·3 episodes per 1000 livebirths), followed by *Ureaplasma* spp (2·4, 1·6–3·2 episodes per 1000 livebirths). Among babies who died, causes were attributed to 46% of pSBI episodes, among which 92% were bacterial. 85 (83%) of 102 blood culture isolates were susceptible to penicillin, ampicillin, gentamicin, or a combination of these drugs.

**Interpretation:**

Non-attribution of a cause in a high proportion of patients suggests that a substantial proportion of pSBI episodes might not have been due to infection. The predominance of bacterial causes among babies who died, however, indicates that appropriate prevention measures and management could substantially affect neonatal mortality. Susceptibility of bacterial isolates to first-line antibiotics emphasises the need for prudent and limited use of newer-generation antibiotics. Furthermore, the predominance of atypical bacteria we found and high incidence of respiratory syncytial virus indicated that changes in management strategies for treatment and prevention are needed. Given the burden of disease, prevention of respiratory syncytial virus would have a notable effect on the overall health system and achievement of Sustainable Development Goal.

**Funding:**

Bill & Melinda Gates Foundation

## Introduction

The Millennium Development Goals led to reductions in deaths among children younger than 5 years by 56%, from 12·7 million in 1990 to 5·6 million, in 2016 worldwide.^1^ In south Asia the reduction was 64%, from 4·8 million to 1·7 million, but the percentage of neonatal deaths increased from 45% in 1990 to 59% in 2016.

The main causes of neonatal deaths in low-income and middle-income countries are prematurity, intrapartum-related events, severe infections, and congenital anomalies.^1^, ^2^, ^3^ Severe infections, including sepsis, meningitis, and pneumonia, are estimated to cause about a third of the 2·6 million neonatal deaths globally or often lead to long-term disabilities.^4^ Prevention of neonatal infections will be necessary to achieve Sustainable Development Goal 3 (ensure healthy lives and promote wellbeing for all at all ages) and the objectives of the Every Woman Every Child initiative.^5^, ^6^ Although neonatal illness is commonly referred to as possible serious bacterial infection (pSBI), the causes remain largely unknown,^7^, ^8^ which is an important impediment to the design of effective prevention and management programmes.

**Research in context**

**Evidence before this study**

The Millennium Development Goals reinforced the global commitment to improve child survival and have led to mortality in children younger than 5 years being lowered by 56% from 12·7 million in 1990 to 5·6 million in 2016. In south Asian countries, mortality in this age group has been reduced by 64%, from 4·8 million to 1·7 million. Neonatal mortality, however, increased from 45% to 59% between 1990 and 2017. We searched PubMed and Ovid, without date restrictions, with 51 combinations of the keywords “neonatal infections”, “South Asia”, “sepsis”, “young infant”, “etiology”, “low income countries”, “clinical algorithm”, and “surveillance”. This search yielded 317 references that were relevant to our research questions. The main causes of neonatal deaths seem to be prematurity, intrapartum complications, severe infection, and congenital anomalies. Severe infection is addressed by treatment and prevention policies focused on clean delivery at birth, breastfeeding, appropriate antibiotic therapy for ill babies, and vaccination. Nevertheless, the causes of most possible serious bacterial infections (pSBIs) remain elusive. In 2012, an estimated 6·9 million neonates worldwide met the WHO definition for pSBI—ie, referral to hospital and antibiotic treatment—although in studies bacteria have been detected by culture in only 5–10% of samples. Information about viral causes of illness is even scarcer. Treatment remains mostly empirical, particularly in developing countries, where most data on antimicrobial resistance derive from hospital-acquired infections. Given the gap in knowledge about causes of pSBI in neonates and the widespread use of treatment with antibiotics, multidrug resistance is a concern. Few studies have included neonates, and even fewer babies on the day of birth, when most neonatal deaths occur. Moreover, studies in low-income and middle-income countries have mostly been hospital based and enrolled babies with pSBI who were seeking health care. In many parts of south Asia, home births are still common and seeking care in the days after birth is rare, even when referred by a community health worker.

**Added value of this study**

We designed the ANISA study to assess unanswered questions specifically in developing countries, in a large sample of pregnant women and babies, by testing blood and respiratory samples from those with symptoms and from healthy controls. We generated population-based evidence on the causes of pSBI through community-wide pregnancy surveillance and enrolment of babies born at home and in health facilities. We included areas with high child mortality and those with state-of-the-art diagnostic services. Stringent protocols were put in place to minimise blood-culture contamination and an external expert panel confirmed classifications of blood culture isolates. As well as blood culture, we incorporated molecular testing in almost 2000 healthy babies and used an innovative partially latent class model to attribute causes to pSBI.

**Implications of all the available evidence**

The list of causal bacteria we identified was long and diverse, making prevention and treatment strategies complex. Respiratory syncytial virus was the leading causal organism in our study, suggesting an opportunity to develop interventions based on maternal immunisation and provision of monoclonal antibodies to neonates. *Ureaplasma* spp had the second leading causal proportion, but have not previously been recognised as common except in very preterm babies, and are not targeted by current recommended antibiotic regimens. Research into the epidemiology and pathogenesis of this group of bacteria in neonates is needed. Despite testing of blood and respiratory samples, for 72% of pSBI episodes, no cause could be attributed. Use of metagenomics approaches, minimally invasive tissue sampling, or both, to investigate causes, with consideration of factors unrelated to infections might facilitate appropriate decisions and investments to save lives among very young babies, limit unnecessary antibiotic use, and curb the emergence of antimicrobial resistance in the community.

Few data are available on the annual number of pSBI episodes in south Asia, despite this region having the highest number worldwide (3·5 million).^2^, ^9^ Determination of the causes of pSBI in neonates has depended on isolation of bacteria by blood culture, which is deemed to be the gold standard test despite poor sensitivity. Two outpatient-based WHO studies done in multiple countries and a population-based study done in low-income and middle-income countries yielded bacterial isolates from less than 10% of babies with pSBI.^10^, ^11^, ^12^ A study in India showed 10% positivity by blood culture, with organisms being predominantly Gram-negative (69%).^13^ A study in Bangladesh examined 62 respiratory samples and found a high burden of viral infections (48%).^14^

Ascertainment of the causes of pSBI has been hindered by several factors: difficulty in case finding (ie, many babies born at home die soon after birth without entering the formal health system); insufficient availability of diagnostic tools to detect bacterial and viral causes; collection of blood samples with inadequate volume, frequent contamination of blood cultures, or both; lack of parallel samples from healthy controls and assessments by sensitive molecular methods to provide information on background colonisation by pSBI pathogens; and absence of appropriate analytical approaches to use information from multiple types of tests and samples to attribute cause. To address these gaps in knowledge, we did the Aetiology of Neonatal Infections in South Asia (ANISA) study in three countries (Bangladesh, India, and Pakistan) to investigate the incidence of pSBI episodes in the first 2 months of life and estimate the proportions of bacterial and viral causes.

## Methods

### Study design and participants

ANISA was an observational cohort study done in five sites in three countries. Four sites were rural (Sylhet, Bangladesh; Matiari, Pakistan; and Vellore and Odisha, India) and one was urban (Karachi, Pakistan). These sites covered a total population of around 1·6 million people, including roughly 250 000 married girls and women of reproductive age (13–49 years) and around 30 000 births per year.^15^ Eligible participants were married women of reproductive age and live babies that could be enrolled up to age 7 days.

We obtained informed verbal consent from pregnant girls and women when they were registered in the study and written informed consent when samples were collected from babies. The study was approved by the ethics committees or internal review boards of all participating organisations.

### Community-based surveillance

We recruited community health workers (CHWs) from the local area through open advertisements. CHWs established active community-based surveillance for pregnancy, pregnancy outcomes, and signs of illness in neonates. Briefly, they followed up married girls and women of reproductive age by home visits to identify pregnancies. They recorded dates of last menstrual period, facilitated or encouraged accessing of antenatal care, built rapport, and arranged visits to assess neonates immediately after birth. Live neonates visited by CHWs within 7 days of birth were registered in the study and scheduled for up to ten home visits up to age 59 days, three week 1 and one per week thereafter. CHWs also collected socioeconomic, household, and maternal information at enrolment and information on labour and delivery at the first visit after birth.

Babies were assessed at every visit for signs of illness, defined as respiratory rate 60 breaths per min or more, severe chest in-drawing, hypothermia (temperature <35·5°C), hyperthermia (temperature ≥38·0°C), movement only with stimulation, convulsions, and poor feeding as reported by the mother and confirmed by CHW assessment.^15^ Babies with one or more signs of pSBI were referred by the CHWs to study-designated health-care facilities or visited at home by a study physician (only in Matiari, Pakistan). To capture illness episodes that occurred between visits, family members were encouraged to seek assessment at a study health facility or via the outreach physician (in Matiari). Ill babies in Bangladesh and Pakistan were examined and managed at primary care facilities or, if they had severe or complicated illness, were referred to tertiary-level hospitals. All ill babies in India were assessed and managed at tertiary-level hospitals.

### Collection of samples

Sample collection has previously been described in detail.^16^ Briefly, trained phlebotomists followed standardised protocols to collect up to 3 mL blood from babies with pSBI episodes for culture and molecular testing. 1 mL blood samples were taken from healthy controls for molecular testing. Strict procedures to minimise contamination during phlebotomy were followed: use of sterile hand gloves and disposible dignity sheets for babies; regular cleaning of the phlebotomy room with disinfectant; and systematic use of antiseptic (alcohol and iodine). Physicians used flocked swabs (Copan Diagnostics, Brescia, Italy) to collect nasopharyngeal and oropharyngeal samples at the health centre from symptomatic babies who met the criteria for pSBI and from healthy babies when scheduled by the study team. To characterise the background presence of potential pSBI pathogens in the community, we used an automated algorithm triggered by CHWs at the first postnatal visit to select randomly registered healthy babies for collection of samples. These babies were assigned an age at which to undergo clinical assessment and sample collection, with assignment probability based on the age distribution of babies with pSBI episodes enrolled to that point.

### Processing of samples

We used automated blood culture (culture media BacTec, BD, Franklin Lakes, NJ, USA or BacT/Alert, Biomérieux, Basinstoke, UK) to isolate bacterial pathogens. Molecular testing was done by PCR with Custom TaqMan Array Cards (Thermo Fisher, Bartlesville, OK, USA) and LifeTech Viia 7 software version 1.2.4 to detect 15 bacterial and 13 viral pathogens.^15^, ^17^ Bacterial strains from the cultures were identified with a conventional biochemical test and the API 20E strip test (bioMérieux-Vitek, Hazelwood, MO, USA), and were confirmed at the Centers for Disease Control and Prevention by matrix-assisted laser desorption/ionisation-time of flight mass spectrometry.^18^ Antimicrobial susceptibility testing was done by disc diffusion, in accordance with Clinical and Laboratory Standards Institute guidelines. We used a MagNA Pure LC total nucleic acid isolation kit (Roche, Basel, Switzerland) to extract nucleic acid from blood samples and nasopharyngeal and oropharyngeal swabs for assessment.

Three infectious disease specialists reviewed and classified each isolate as a pathogen or as not clinically relevant, based on the clinical features of the patient, drug susceptibility, illness management, and outcome.^19^ 10% of molecular samples were retested at the Centers for Disease Control and Prevention to provide quality control and assurance for site results (appendix). As an additional assessment, we used real-time PCR for speciation of *Bordetella* spp and *Ureaplasma* spp and confirmation of *Escherichia coli* positivity.

### Definition of pSBI episodes

We defined confirmed pSBI episodes as the presence of one sign of illness assessed by the CHWs, no hospital admission in the preceding 7 days (except for postnatal hospital stays for babies born in health facilities), and no registered pSBI episode in the preceding 7 days. We excluded babies with increased respiratory rate as the only sign because, during the study period, independent evidence showed low specificity for this sign alone^20^ and samples were not collected in several sites. Test results for samples from healthy babies were included if the criteria for pSBI were not met on the day of assessment or in the previous 7 days before and after sample collection.

### Statistical analysis

We aimed to enrol 400 healthy babies in each site in Bangladesh and Pakistan and 200 in each Indian site.^21^ The sample size was constrained by ethical and community acceptability, but we calculated that the target sample size would be sufficient to provide initial estimation of background colonisation with set false-positive rates (appendix) and a maximum SE of 3%.

The primary objective was to estimate the proportion of pSBI episodes attributed to each pathogen assessed. Because the study design included two types of sample (blood and respiratory), up to three tests per pathogen (blood culture, blood molecular assay, and respiratory molecular assay), and because each of these tests had its own sensitivity and specificity for detection of the true cause of the pSBI episode, we used a partially latent class model, which was originally developed for a similarly designed study of the causes of pneumonia^22^ and adapted to meet the ANISA study specifications (appendix). For all babies with pSBI episodes and healthy babies with at least one laboratory test result, we included blood culture final determination data (for those with pSBI episodes) and observed binary molecular assay results (positive or negative) in the model input dataset. We attributed causal proportions to 28 target pathogens and two additional classes: other blood culture (all organisms isolated from blood cultures that did not have an associated pathogen-specific molecular assay test) and other or none (episodes that could not be attributed to any pathogens).

The primary output of the model was mean pathogen proportions at the population level with 95% credible intervals (CrIs), from which individual-level proportions could be derived (appendix). Covariates were study site, age at onset, date of enrolment, and outcome status (died but had a sample available from the previous 7 days *vs* alive, except for Vellore, where there were too few deaths to stratify by outcome). We ran the model under various input dataset conditions, for which performance was assessed by simulation studies (appendix). Model convergence was assessed through trace and other diagnosis plots. We assessed model assumptions with interim model outputs by stopping the Gibbs sampler at random cycles. Model fit to input data was evaluated by comparing the fitted and observed numbers of positives for each blood molecular assay and blood culture. We did programming and computation with R (version 3.2.5), SAS (version 9.3), and Stata (version 13).

### Role of the funding source

The funder of the study had no role in study design, data collection, data analysis, data interpretation, or writing of the report. The corresponding author had access to all data in the study and had responsibility for the decision to submit for publication.

## Results

**Figure 1:**
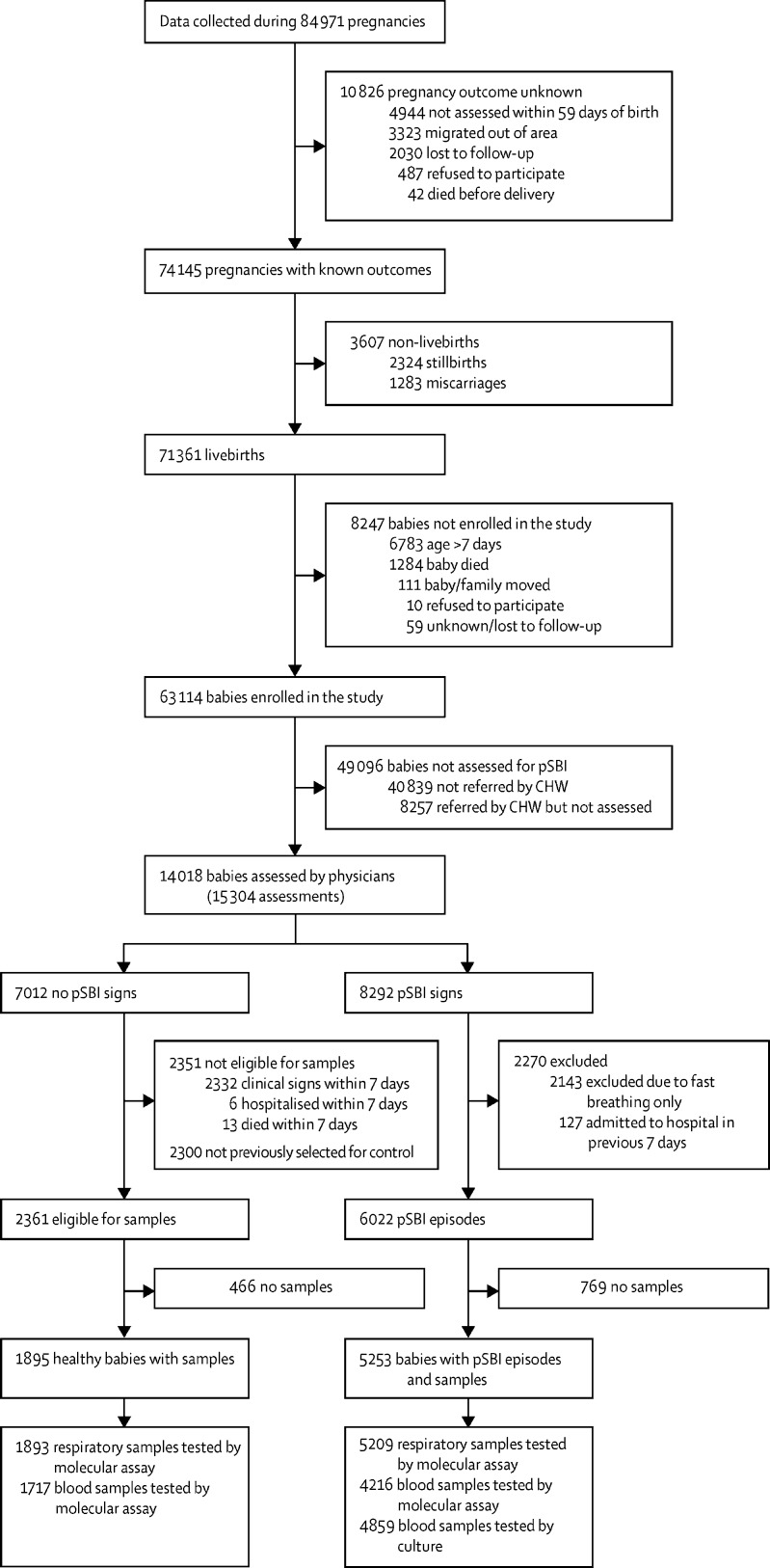
Study flow diagram pSBI=possible serious bacterial infection. CHW=community health worker.

During surveillance, pregnancy outcome data were collected for 74 145 (87%) of 84 971 pregnancies. 71 361 were livebirths, 2324 were stillbirths, and 1283 were miscarriages. 63 114 (88%) liveborn babies were registered and followed up for 59 days, and the remaining 8247 babies could not be reached within 7 days, due to death, migration out of the area, or refusal to participate in the study (figure 1, appendix). 56 960 (90%) liveborn neonates were recruited within 72 h. Population characteristics, in particular socioeconomic status, home delivery, pregnancy outcomes, and early death among babies, varied by site (table 1). 43 665 (69%) registered babies received all three visits in the first week, and 48 415 (77%) received at least eight of ten scheduled visits by CHWs. These visits resulted in 12 836 referrals for clinical assessment by physicians, of which 10 809 (84%) were attended. Caregivers made 3683 additional self-referrals.

**Table 1:** Characteristics of mothers with a birth outcome and of registered liveborn babies

		**Sylhet**	**Karachi**	**Matiari**	**Odisha**	**Vellore**	**Total**
**Maternal**							
All		22 426	17 700	19 251	8424	6344	74 145
Age at delivery (years)		26 (13–53)	26 (13–55)	29 (13–54)	25 (14–49)	24 (14–44)	27 (13–55)
First birth		6678 (32%)	3619 (25%)	3414 (22%)	4050 (51%)	2724 (50%)	20 485 (28%)
Stillbirths (per 1000 pregnancies with known outcome)		35·3	30·1	39·0	19·2	9·9	31·0
Miscarriages/spontaneous abortion (per 1000 pregnancies with known outcome)		12·9	28·2	18·1	6·3	14·5	17·3
Poor nutritional status*		3677 (16%)	1133 (7%)	1351 (7%)	774 (9%)	374 (6%)	7309 (10%)
Received full antenatal package†		6778 (64%)	6973 (57%)	5729 (42%)	6041 (93%)	4319 (68%)	29 840 (40%)
At least one antenatal care visit with a skilled provider		13 518 (99%)	12 878 (95%)	16124 (99%)	7912 (98%)	6326 (100%)	56 758 (77%)
Birth location							
	Health facility	3744 (17%)	9709 (56%)	12 502 (66%)	8001 (96%)	6230 (100%)	40 186 (54%)
	Home	18 322 (83%)	7478 (43%)	6379 (34%)	368 (4%)	21 (<1%)	32 568 (44%)
Skilled birth attendant‡		4355 (20%)	10 381 (60%)	12 410 (66%)	78 04 (93%)	6234 (100%)	41 184 (56%)
Clean delivery kit		2814 (13%)	13 358 (78%)	13 133 (69%)	7536 (90%)	6112 (98%)	42 953 (58%)
Ever attended school/madrasa		17 223 (77%)	8375 (49%)	3340 (17%)	6856 (81%)	6227 (98%)	42 021 (57%)
Household members		6 (1–35)	8 (1–35)	7 (1–35)	5 (1–31)	4 (2–29)	6 (1–35)
Electricity		10 127 (45%)	16 763 (99%)	17 373 (90%)	6945 (82%)	6315 (100%)	57 523 (78%)
Piped water		271 (1%)	10 677 (63%)	5667 (29%)	1235 (15%)	5414 (85%)	23 264 (31%)
Mobile phone ownership		18 373 (82%)	13 069 (77%)	14 123 (73%)	7245 (86%)	6006 (95%)	58 816 (80%)
**Babies**							
All		19 007	13 321	16 462	8197	6127	63 114
Age at registration (h)		6 (0–60)	20 (0–162)	18 (0–159)	7 (0–156)	7 (0–143)	12 (0–162)
Child-days observed (in 10 000 days)		101·6	57·5	76·6	43·2	34·4	313·5
Boys/girls		9669 (51%)/9338 (49%)	6812 (51%)/6509 (49%)	8516 (52%)/7946 (48%)	4247 (52%)/3950 (48%)	3175 (52%)/2952 (48%)	32 419 (51%)/30 695 (49%)
Preterm		3336 (18%)	2485 (20%)	4882 (30%)	1341 (17%)	431 (7%)	12 475 (20%)
Low birthweight		5153 (27%)	3116 (24%)	6191 (38%)	1593 (19%)	779 (13%)	16 832 (27%)
Massage after birth		3053 (16%)	6249 (47%)	11 379 (69%)	2023 (25%)	0	22 704 (36%)
Washed after birth		2613 (14%)	12 634 (95%)	16 130 (98%)	6411 (78%)	3922 (64%)	41 710 (66%)
Proper cord care at birth§		980 (5%)	3711 (22%)	6817 (37%)	1378 (17%)	6034 (97%)	18 920 (27%)
Age at breastmilk supplementation (days)¶		35 (0–62)	4 (0–65)	6 (0–65)	4 (0–60)	27 (0–65)	18 (0–35)
Ever vaccinated (assessed at day 59 visit)		6790/16 261 (42%)	5035/82 00 (61%)	4466/6563 (68%)	6581/7064 (93%)	5767/5814 (99%)	28 639/43 902 (65%)
	BCG	4003 (59%)	3916 (78%)	4466 (100%)	5960 (91%)	5474 (95%)	23 819 (83%)
	At least one oral polio vaccine	1937 (29%)	3439 (68%)	3845 (86%)	4930 (75%)	5461 (95%)	19 612 (68%)
	Diphtheria, tetanus, and pertussis toxin	1430 (22%)	904 (18%)	1829 (41%)	3671 (56%)	5053 (88%)	12 887 (45%)
	Pneumococcal conjugate vaccine	50 (1%)	119 (2%)	2 (<1%)	113 (2%)	20 (<1%)	304 (1%)
Mean early onset of infection per 1000 livebirths (95% CI)		33·0 (30·5–35·7)	51·8 (48·1–55·9)	44·3 (41·2–47·7)	25·7 (22·5–29·5)	38·8 (34·2–44·1)	39·6 (38·1–41·2)
Mean late onset of infection per 1000 livebirths (95% CI)		53·7 (50·5–57·1)	60·7 (56·7–65·1)	51·1 (47·8–54·7)	74·4 (68·7–80·5)	39·4 (34·8–44·8)	55·8 (54·0–57·7)
Mean mortality among babies younger than 60 days per 1000 livebirths (95% CI)		46 (44–48)	48 (45–52)	57 (54–61)	17 (14–20)	11 (8–14)	43 (41–45)

Data are median, median (range), or n (%), unless stated otherwise. Variables with ≥10% missing data were number of antenatal care visits (28%), first birth (13%), and materials used to cut and tie the umbilical cord (13%).

* Defined as mid-upper-arm circumference <21·5 cm.

† Receipt of at least two antenatal visits from a community health worker with receipt of two tetanus injections during current pregnancy, one tetanus injection and at least four shots before pregnancy, or at least five tetanus injections before pregnancy, and receipt of at least one iron tablet or dose of iron syrup during current pregnancy.

‡ Qualified doctor, nurse, midwife, or paramedic.

§ For cutting, boiled thread, clip kit, knife, blade, or tongs in the home or nurse's or doctor's scissor or clip kit in a hospital or health facility; for tying, boiled clip, clip kit, thin rope brought by doctor, blade, or rubber band followed by application of antibiotic or antiseptic to the stump in the home, and clip or clip kit followed by application of antibiotic, antiseptic, or nothing in the hospital of health facility.

¶ Age at which any food or liquid other than breastmilk was first given to the baby.

Physicians identified 6022 pSBI episodes, including 2498 early-onset (<3 days) and 3524 late-onset episodes (3–59 days). 289 (5%) babies had more than one episode (figure 1, table 2, appendix). The overall pSBI incidence was 95·4 episodes per 1000 livebirths (range 78·3 per 1000 in Vellore, India, to 112·6 per 1000 in Karachi, Pakistan) and was notably higher in the first 3 days of life than in the remaining follow-up period (55·8 per 1000 livebirths *vs* 39·6 per 1000 livebirths), which is equivalent to an increase in incidence density rate of roughly 13·5 times.

**Table 2:** Characteristics of possible serious bacterial infection episodes

			**Sylhet**	**Karachi**	**Matiari**	**Odisha**	**Vellore**	**Total**
**Maternal**								
All			1540	1401	1481	782	446	5650
Age			26 (14–53)	26 (14–52)	30 (15–54)	24 (15–43)	24 (16–39)	27 (14–54)
First livebirth			543 (35%)	340 (24%)	354 (24%)	421 (54%)	275 (62%)	1933 (34%)
Ever attended school/madrasa			1106 (72%)	603 (44%)	191 (13%)	643 (82%)	436 (98%)	2979 (53%)
At least one antenatal care visit with skilled provider			832 (54%)	1009 (72%)	1258 (85%)	738 (94%)	446 (100%)	4283 (76%)
Birth location								
	Health facility		164 (11%)	717 (51%)	957 (65%)	753 (96%)	444 (100%)	3029 (54%)
	Home		1376 (89%)	684 (49%)	524 (35%)	29 (4%)	2 (<1%)	2610 (46%)
Skilled birth attendant*			216 (14%)	775 (55%)	945 (64%)	726 (93%)	444 (100%)	3106 (55%)
Clean delivery kit			159 (10%)	1083 (77%)	1015 (68%)	706 (90%)	437 (98%)	3400 (60%)
Household members			6 (2–27)	8 (2–35)	7 (2–35)	5 (2–24)	4 (2–19)	6 (2–35)
**Babies**								
All			1561	1416	1491	790	455	5713
Boys/girls			909 (58%)	785 (55%)	808 (54%)	467 (59%)	272 (60%)	3241 (57%)
Preterm			447 (29%)	361 (27%)	499 (34%)	182 (23%)	75 (17%)	1564 (28%)
Low birthweight			619 (43%)	455 (36%)	605 (45%)	230 (30%)	93 (21%)	2002 (38%)
Ever vaccinated (assessed at day 59 visit)			494/1151 (43%)	462/818 (56%)	528/1051 (50%)	627/685 (92%)	426/433 (98%)	2537/4138 (61%)
	BCG		279 (24%)	345 (42%)	365 (35%)	578 (84%)	405 (94%)	1972 (48%)
	At least one oral polio vaccine		207 (18%)	298 (36%)	310 (30%)	508 (74%)	406 (94%)	1729 (42%)
	Diphtheria, tetanus, and pertussis toxin		105 (9%)	72 (9%)	135 (13%)	369 (54%)	355 (82%)	1036 (25%)
	Pneumococcal conjugate vaccine		3 (<1%)	7 (1%)	0	10 (2%)	2 (1%)	22 (1%)
Possible serious bacterial infection episodes			1649	1500	1572	821	480	6022
	Early onset (age <3 days)		628 (38%)	691 (46%)	730 (46%)	211 (26%)	238 (50%)	2498 (42%)
Integrated management of childhood infection signs								
	Respiratory rate ≥60 breaths per min		756 (46%)	600 (40%)	681 (43%)	175 (21%)	98 (20%)	2310 (38%)
	Severe chest in-drawing		602 (37%)	385 (26%)	290 (18%)	58 (7%)	83 (17%)	1418 (24%)
	Axillary temperature ≥38·0°C		326 (20%)	467 (31%)	789 (50%)	372 (45%)	96 (20%)	2050 (34%)
	Axillary temperature <35·5°C		284 (17%)	196 (13%)	182 (12%)	27 (3%)	8 (2%)	697 (12%)
	Movement only when stimulated or no movement		299 (18%)	164 (11%)	161 (10%)	223 (27%)	72 (15%)	919 (15%)
	Convulsions (observed or in history)		306 (19%)	88 (6%)	299 (19%)	65 (8%)	17 (4%)	775 (13%)
	Poor feeding (confirmed by observation)		702 (43%)	557 (37%)	363 (23%)	596 (73%)	301 (63%)	2519 (42%)
	More than one sign		1090 (66%)	769 (51%)	904 (58%)	476 (58%)	157 (33%)	3396 (56%)
Other clinical signs								
	Umbilical flare or infection		28 (2%)	20 (1%)	43 (3%)	12 (3%)	22 (3%)	125 (2%)
	Nappy rash or skin pustules		83 (5%)	98 (7%)	69 (4%)	102 (12%)	28 (6%)	380 (6%)
	Conjunctivitis		54 (3%)	7 (<1%)	12 (1%)	29 (4%)	5 (1%)	107 (2%)
	Bulging fontanelle		13 (1%)	2 (<1%)	27 (2%)	7 (1%)	1 (<1%)	50 (1%)
	Hospital admission		417 (25%)	193 (13%)	129 (8%)	563 (69%)	441 (92%)	1743 (29%)
	Death		307 (19%)	169 (11%)	220 (14%)	52 (6%)	15 (3%)	763 (13%)
		Within 7 days of episode	239 (15%)	116 (8%)	149 (10%)	40 (5%)	8 (2%)	552 (9%)

Data are n, n (%), or median (IQR).

* Qualified doctor, nurse, midwife, or paramedic.

Blood samples were collected from 4859 (81%) of 6022 babies during pSBI episodes, of which all were tested by blood culture and 4216 (70%) were tested by molecular assay (figure 1). 602 (10%) babies had received antibiotics before blood samples were collected, mainly in Matiari, Pakistan (439 [73%]). Molecular test results for respiratory samples were available for 5209 (87%) of 6022 babies with pSBI episodes. We also collected 1726 blood samples (results available for 1717) and 1893 respiratory samples from 1895 healthy babies for molecular testing (figure 1). Healthy babies were older than sick children at the time of sample collection (median age 13·4 days, IQR 2·8–33·8 *vs* 5·7 days, 1·4–27·4) and cumulative enrolment of controls did not always track pSBI episodes (appendix).

Of 4859 pSBI blood cultures, 338 (7%) yielded bacteria, of which 206 were judged not to be clinically relevant based on predefined criteria, 30 not to be clinically relevant by an independent review panel, and 102 to be clinically relevant. The incidence of culture-confirmed infection was 1·6 per 1000 livebirths. Causal organisms in blood cultures were predominately *E coli, Klebsiella* spp, *Staphylococcus aureus,* and group A streptococcus. More than half of causal bacteria were Gram negative, led by *Klebsiella* spp and *E coli* (table 3), and incidence of Gram-negative organisms was higher among hospital-born babies than in community-born babies (1·3 per 1000 livebirths *vs* 0·7 per 1000 livebirths). Most isolates were susceptible to penicillin, ampicillin, gentamicin, or a combination of these drugs (table 3). Isolates were more susceptible to gentamicin (75 [74%]) than ampicillin or penicillin (27 [41%]).

**Table 3:** Characteristics of clinically relevant bacteria isolated in blood cultures from babies with possible serious infections

		**No.**	**Age <72 h at culture (%)**	**Boys/girls (%)**	**Place of birth (%)**		Gestational age <37 weeks*	**Died**	**Site**					**Susceptibility to penicillin, ampicillin, and/or gentamicin**†
					Hospital	Home			Sylhet	Karachi	Matiari	Vellore	Odisha	
Gram positive														
	All	36	10 (28)	13 (36%)/23 (64%)	17 (47%)	19 (53%)	7 (19%)	7 (19%)	15 (42%)	2 (6%)	7 (19%)	5 (14%)	7 (19%)	35 (97%)
	*Enterococcus faecium*	1	0	0	1 (100%)	0	0	1 (100%)		0	0	0	1 (100%)	..
	Group A streptococcus	11	1 (9%)	2 (18%)/9 (82%)	5 (45%)	6 (55%)	1 (9%)	0	4 (36%)	2 (18%)	4 (36%)	1 (9%)	0	11 (100%)
	Group B streptococcus	6	6 (100%)	3 (50%)/3 (50%)	1 (17%)	5 (83%)	3 (50%)	2 (66%)	5 (83%)	0	0	1 (17%)	0	6 (100%)
	*Staphylococcus aureus*	12	0	5 (42%)/7 (58%)	10 (83%)	2 (17%)	2 (17%)	1 (8%)	1 (8%)	0	2 (17%)	3 (25%)	6 (50%)	11 (92%)
	*Streptococcus oralis*	1	0	1 (100%)/0	0	1 (100%)	0	1 (100%)	1 (100%)	0	0	0	0	..
	*Streptococcus pneumoniae*	5	3 (60%)	2 (40%)/3 (60%)	0	5 (100%)	1 (20%)	2 (40%)	4 (80%)	0	1 (20%)	0	0	5 (100%)
Gram negative														
	All	66	24 (36%)	38 (58%)/28 (42%)	47 (71%)	19 (29%)	21 (32)	23 (35)	12 (18%)	13 (20)	10 (15)	5 (8%)	26 (39%)	50 (75%)
	*Acinetobacter* spp‡	6	3 (50%)	6 (100%)/0	4 (66%)	2 (33%)	1 (17%)	1 (17%)	0	2 (33%)	1 (17%)	0	3 (50%)	3 (50%)
	*Burkholderia cepacia*	2	1 (50%)	1 (50%)/1 (50%)	2 (100%)	0	0	0	0	0	2 (100%)	0	0	..
	*Citrobacter koseri*	1	1 (100%)	0/1 (100%)	1 (100%)	0	0	0	0	0	0	0	1 (100%)	..
	*Edwardsiella tarda*	1	0	1 (100%)/0	0	1 (100%)	0	0	1 (100%)	0	0	0	0	..
	*Enterobacter* spp§	3	0	2 (66%)/1 (33%)	3 (100%)	0	1 (33%)	1 (33%)	0	1 (33%)	0	1 (33%)	1 (33%)	..
	*Escherichia coli*¶	21	9 (43%)	11 (52%)/10 (48%)	16 (76%)	5 (24%)	10 (48%)	11 (52%)	4 (19%)	3 (14%)	3 (14%)	3 (14%)	8 (38%)	16 (75%)
	*Klebsiella* spp‖	17	6 (86%)	10 (59%)/7 (41%)	16 (94%)	1 (6%)	3 (18%)	4 (24%)	1 (6%)	2 (12%)	2 (12%)	1 (6%)	11 (65%)	13 (75%)
	*Morganella morganii*	1	0	0/1 (100%)	0	1 (100%)	0	1 (100%)	1 (100%)	0	0	0	0	..
	Neisseria meningitidis**	5	0	3 (60%)/2 (40%)	1 (20%)	4 (80%)	1 (20%)	0	4 (80%)	1 (20%)	0	0	0	5 (100%)
	*Proteus mirabilis*	1	1 (100%)	0/1 (100%)	0	1 (100%)	1 (100%)	0	0	1 (100%)	0	0	0	..
	*Pseudomonas* spp††	3	1 (33%)	1 (33%)/2 (66%)	1 (33%)	2 (66%)	1 (33%)	3 (100%)	1 (33%)	0	2 (66%)	0	0	..
	*Salmonella enterica*	2	0	1 (50%)/1 (50%)	0	2 (100%)	1 (50%)	0	0	2 (100%)	0	0	0	..
	*Serratia marcescens*	3	2 (66%)	2 (66%)/1 (33%)	3 (100%)	0	2 (66%)	2 (66%)	0	1 (33%)	0	0	2 (66%)	..
Total		102	34 (33%)	51 (50%)/51 (50%)	64 (63%)	38 (37%)	28 (27%)	30 (29%)	27 (26%)	15 (15%)	17 (17%)	10 (10%)	33 (32%)	85 (83%)

* Two babies had missing values for gestational age.

† WHO recommended treatment for neonatal sepsis at community level; individual percentages were calculated for the organisms associated with five or more episodes of infection.

‡ *A baumanii* (n=3), *A lwoffii* (n=2), *A schindleri* (n=1); and *A baumanii* isolated in a polymicrobial culture with *Serratia marcescens*.

§ *E aerogenes* (n=1) and *E cloacae* (n=2).

¶ *E coli* found in two polymicrobial cultures (one with *Enberobacter cloacae* and one with *A baumanii*).

‖ *K pneumoniae* (n=16); *K terrigena* (n=1); and *K pneumoniae* found in one polymicrobial culture with *Pseudomonas aeruginosa*.

** *N meningitidis* found in one polymicrobial culture with *Plesiomonas shigelloides*.

†† *P aeruginosa* (n=1); *P fluorescens* (n=1); *P pseudomallei* (n=1).

Among pSBI blood samples tested by molecular assay, 487 (12%) of 4216 tested positive for at least one target, led by enterovirus or rhinovirus, *Salmonella* spp, *Klebsiella* spp, and *E coli*, and 57 (1%) were positive for multiple targets. Among 5209 respiratory samples 3907 (75%) were positive for at least one target and 1302 (47%) were positive for multiple targets (table 4). The numbers of positive results were similar for blood and respiratory samples from healthy babies, with some variation by site, age, and outcome (table 4, appendix). Pathogens were more prevalent among babies with pSBI than among healthy babies, including for respiratory syncytial virus and *Ureaplasma* spp (table 4). Among 566 samples containing *Ureaplasma* spp, 311 (55%) were *U parvum*, 255 (45%) were *U urealyticum*, and the remainder contained both species. *Bordetella* spp were detected in similar proportions of babies with pSBI and healthy babies, with most samples containing these species seen in Karachi, Pakistan (table 4). Speciation of *Bordetella* spp revealed that 84 (36%) of 234 samples from babies with pSBI and 30 (41%) of 73 from healthy babies contained *B pertussis*.

**Table 4:** Detected pathogens among babies with possible serious infections and healthy babies

	**Nasopharyngeal or oropharyngeal**			**Blood**		
	Positive babies with symptoms (n=5209)	Positive healthy babies (n=1893)	Odds ratio (95% CI)	Positive babies with symptoms (n=4216)	Positive healthy babies (n=1717)	Odds ratio (95% CI)
**Bacteria**						
*Bordetella* spp	234 (5%)	73 (4%)	1·2 (0·9–1·5)	..	..	..
*Chlamydia pneumoniae*	11 (<1%)	3 (<1%)	1·3 (0·4–4·8)	..	..	..
*Chlamydia trachomatis*	12 (<1%)	5 (<1%)	0·9 (0·3–2·5)	..	..	..
*Escherichia coli*	1363 (26%)	461 (24%)	1·1 (1·0–1·2)	75 (2%)	32 (2%)	1·0 (0·6–1·5)
Group A streptococcus	..	..	..	14 (<1%)	0	
Group B streptococcus	427 (8%)	123 (7%)	1·3 (1·0–1·6)	19 (1%)	6 (<1%)	1·3 (0·5–3·2)
Pan*-Haemophilus influenzae*	..	..	..	36 (1%)	6 (<1%)	2·5 (1·0–5·8)
*Klebsiella pneumoniae*	1149 (22%)	422 (22%)	1·0 (0·9–1·1)	77 (2%)	33 (2%)	1·0 (0·6–1·4)
*Mycoplasma pneumoniae*	10 (<1%)	1 (<1%)	3·6 (0·5–28·5)	..	..	..
*Neisseria meningitidis*	..	..	..	7 (<1%)*	4 (<1%)	0·7 (0·2–2·5)
*Pseudomonas aeruginosa*	..	..	..	19 (1%)	7 (<1%)	1·1 (0·5–2·6)
*Salmonella* spp	..	..	..	78 (2%)	30 (2%)	1·1 (0·7–1·6)
*Staphylococcus aureus*	..	..	..	29 (1%)	15 (1%)	0·8 (0·4–1·5)
*Streptococcus pneumoniae*	1843 (35%)	628 (33%)	1·1 (1·0–1·2)	61 (2%)	20 (1%)	1·3 (0·8–2·1)
*Ureaplasma* spp	566 (11%)	118 (6%)	1·8 (1·5–2·3)	19 (1%)	6 (<1%)	1·3 (0·5–3·2)
**Viruses**						
Adenovirus	75 (1%)	34 (2%)	0·8 (0·5–1·2)	..	..	..
Cytomegalovirus	363 (8%)†	153 (9%)‡	0·9 (0·7–1·1)	..	..	..
Enterovirus or rhinovirus	1521 (29%)	644 (34%)	0·8 (0·7–0·9)	131 (3%)	49 (3%)	1·1 (0·8–1·5)
Human metapneumovirus	21 (<1%)	6 (<1%)	1·3 (0·5–3·2)	..	..	..
Human parechovirus	20 (<1%)	12 (1%)	0·6 (0·3–1·2)	..	..	..
Influenza type A	53 (1%)	13 (1%)	1·5 (0·8–2·7)	..	..	..
Influenza type B	31 (1%)	3 (<1%)	3·8 (1·2–12·4)	..	..	..
Parainfluenza 1	25 (1%)	7 (<1%)	1·3 (0·6–3·0)	..	..	..
Parainfluenza 2	7 (<1%)	7 (<1%)	0·4 (0·1–1·0)	..	..	..
Parainfluenza 3	65 (1%)	16 (1%)	1·5 (0·9–2·6)	..	..	..
Respiratory syncytial virus	402 (8%)	25 (1%)	6·3 (4·2–9·4)	..	..	..
Rubella	20 (<1%)	5 (<1%)	1·5 (0·6–3·9)	..	..	..

* Of 4195 tested.

† Of 4751 tested.

‡ Of 1800 tested.

**Figure 2:**
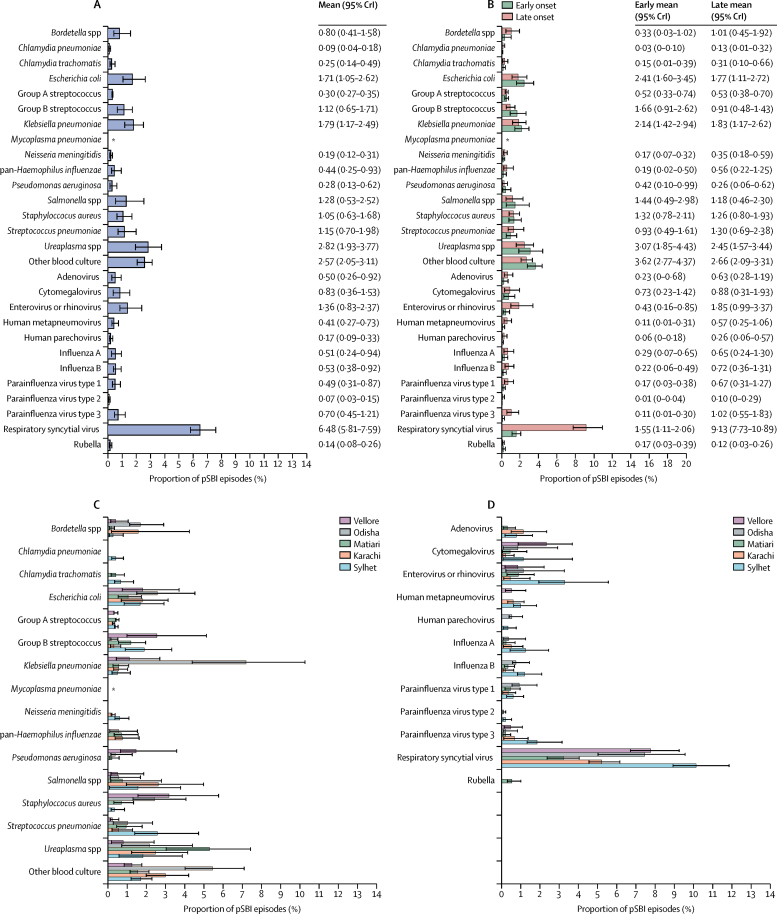
Attributed proportions of causal pathogens tested during possible serious bacterial infection episodes, estimated in a partially latent class model (A) Estimates from overall model. (B) Estimates by age of onset. (C) Estimates of bacterial causes by study site. (D) Estimates of viral causes by study site. CrI=credible interval. pSBI=possible serious bacterial infection. *None detected.

The estimated test-specific true-positive and false-positive rates underlying the model-based proportions of causal organisms and the estimated incidence are shown in the appendix. Overall, 16% and 12% of pSBI episodes were attributed to bacteria and viruses, respectively, and the remaining 72% could not be attributed to any of the tested pathogens. The leading pathogen was respiratory syncytial virus, seen in 6·5% of cases (95% CrI 5·8–7·6), followed by *Ureaplasma* spp, seen in 2·8% (1·9–3·8; figure 2). *Klebsiella pneumoniae, E coli*, enterovirus or rhinovirus, *Salmonella* spp, *Streptococcus pneumoniae*, group B streptococcus, and *S aureus* were each attributed as the causal agent in more than 1·0% of pSBI episodes (figure 2). Attribution of cause to a specific pathogen was less likely among early-onset than among late-onset episodes (22·5%, 95% CrI 19·2–26·0 *vs* 33·1%, 29·8–36·8). Furthermore, among early-onset episodes, bacterial attributions were much more frequent than viral (18·4% *vs* 4·1%; figure 2). *Ureaplasma* spp were the most frequent causal organisms, with an estimated pathogen proportion of 3·1% (95% CrI 1·9–4·4). *E coli, K pneumoniae*, group B streptococcus, *Salmonella* spp, *S aureus,* and respiratory syncytial virus all had proportion point estimates greater than 1·0% (figure 2). Among late-onset episodes, the proportion attributed to viruses was 16·5%, similar to that attributed to bacteria (16·6%) and were predominantly respiratory syncytial virus (9·1%, 95% CrI 7·7–10·9) and *Ureaplasma* spp (2·5%, 1·6–3·4; figure 2). Attribution of bacterial causes differed by site: the leading bacterial causes were *S pneumoniae* in Sylhet (2·6% of episodes, 95% CrI 1·4–4·7), *Ureaplasma* spp in Matiari (5·3%, 3·0–7·4), and *Klebsiella* spp in Odisha (7·2%, 4·4–7·2; figure 2). Respiratory syncytial virus was the leading viral cause in all sites (figure 2).

The overall incidence of bacterial infections was 13·2 (95% CrI 11·2–15·6) per 1000 livebirths (table 5). For *Ureaplasma* spp, the overall incidence was 2·4 (95% CrI 1·6–3·2) per 1000 livebirths, with the highest incidence seen in Matiari. *K pneumoniae* was the next leading pathogen, for which the highest incidence was in Odisha. Incidence of *E coli* varied little across sites and *S aureus* was a rare cause except in the Indian sites, where incidence was greater than 2·0 per 1000 livebirths (table 5).

**Table 5:** Estimated cause-specific incidence per 1000 livebirths, based on results of a partially latent class model

	Overall*	**Sylhet**	**Karachi**	**Matiari**	**Vellore**	**Odisha**
**Bacteria**†						
All bacteria tested	13·24 (11·18–15·63)	10·38 (7·69–13·62)	13·18 (9·3–17·91)	11·43 (8·78–14·32)	10·61 (7·44–14·89)	23·46 (18·52–29·27)
*Bordetella* spp	0·66 (0·34–1·32)	0·19 (0·04–0·57)	1·48 (0·17–3·98)	0·14 (0·07–0·27)	0·3 (0·11–0·82)	1·63 (1·1–2·82)
*Chlamydia pneumoniae*	0·07 (0·04–0·15)	0·29 (0·14–0·58)	0	0	0	0
*Chlamydia trachomatis*	0·21 (0·12–0·41)	0·48 (0·25–0·96)	0	0·33 (0·14–0·7)	0	0
*Escherichia coli*	1·43 (0·88–2·18)	1·21 (0·6–2·11)	1·68 (0·65–2·94)	0·86 (0·43–1·42)	1·42 (0·58–2·89)	2·49 (1·46–4·4)
Group A streptococcus	0·25 (0·23–0·29)	0·28 (0·24–0·38)	0·25 (0·22–0·33)	0·36 (0·31–0·47)	0·25 (0·24–0·4)	0
Group B streptococcus	0·93 (0·54–1·43)	1·36 (0·65–2·4)	0·28 (0·12–0·62)	0·97 (0·45–1·61)	1·99 (0·78–4·01)	0·16 (0·1–0·5)
Pan-*Haemophilus influenzae*	0·38 (0·21–0·79)	0	0·69 (0·35–1·52)	0·57 (0·26–1·29)	0	0·53 (0·08–1·49)
*Klebsiella pneumoniae*	1·49 (0·97–2·06)	0·36 (0·15–0·83)	0·52 (0·28–0·96)	0·46 (0·22–0·89)	0·87 (0·34–2·11)	6·99 (4·27–9·99)
*Mycoplasma pneumoniae*	0 (0–0)	0	0	0	0	0
*Neisseria meningitidis*	0·15 (0·1–0·26)	0·44 (0·26–0·79)	0·2 (0·15–0·35)	0	0	0
*Pseudomonas aeruginosa*	0·24 (0·11–0·52)	0	0	0·17 (0·08–0·47)	1·14 (0·5–2·8)	0·35 (0·14–1·23)
*Salmonella* spp	1·07 (0·44–2·11)	1·12 (0·06–2·73)	2·46 (0·9–4·69)	0·61 (0·11–2·28)	0·39 (0·11–1·46)	0·54 (0·12–1·64)
*Staphylococcus aureus*	0·88 (0·53–1·4)	0·25 (0·12–0·62)	0	0·57 (0·25–1·1)	2·49 (1·22–4·51)	2·35 (1·26–3·95)
*Streptococcus pneumoniae*	0·96 (0·58–1·64)	1·85 (1·01–3·41)	0·51 (0·19–1·2)	0·77 (0·37–1·46)	0·17 (0·09–0·44)	0·99 (0·26–2·25)
*Ureaplasma* spp	2·38 (1·62–3·17)	1·32 (0·42–2·79)	2·32 (1·16–3·9)	4·34 (2·49–6·1)	0·62 (0·14–1·88)	2·1 (0·69–4·27)
Other in blood culture‡	2·14 (1·71–2·6)	1·24 (0·87–1·66)	2·81 (1·87–3·95)	1·27 (0·94–1·75)	0·97 (0·66–1·37)	5·3 (3·91–6·9)
**Viruses**†						
All viruses tested	10·13 (9·04–11·55)	15·68 (13·41–18·52)	8·7 (7·14–10·78)	5·46 (4·29–6·87)	9·31 (7·75–11·25)	11·81 (8·68–15·35)
Adenovirus	0·42 (0·22–0·77)	0·56 (0·13–1·16)	1·06 (0·47–2·21)	0·24 (0·06–0·59)	0	0
Cytomegalovirus	0·69 (0·3–1·27)	0·82 (0·05–2·67)	0·19 (0·05–0·62)	0·36 (0·04–1·07)	1·82 (0·67–2·88)	0·82 (0·08–2·84)
Enterovirus or rhinovirus	1·12 (0·69–1·96)	2·37 (1·4–4·02)	0·41 (0·11–1·4)	0·71 (0·23–1·39)	0·66 (0·14–1·73)	1·09 (0·21–3·18)
Human metapneumovirus	0·34 (0·22–0·61)	0·72 (0·45–1·31)	0·56 (0·3–1·1)	0	0·4 (0·16–0·99)	0
Human parechovirus	0·14 (0·07–0·27)	0·24 (0·06–0·55)	0	0	0	0·52 (0·37–1·06)
Influenza type A	0·42 (0·2–0·78)	0·89 (0·32–1·77)	0·47 (0·09–1·05)	0·19 (0·06–0·57)	0	0·34 (0·07–1·21)
Influenza type B	0·44 (0·31–0·76)	0·86 (0·6–1·51)	0·18 (0·07–0·59)	0·27 (0·11–0·55)	0	0·72 (0·56–1·41)
Parainfluenza 1	0·41 (0·26–0·72)	0·43 (0·19–0·82)	0·32 (0·06–0·69)	0·37 (0·15–0·78)	0	0·89 (0·52–1·79)
Parainfluenza 2	0·06 (0·03–0·13)	0·14 (0·05–0·38)	0 (0–0)	0·07 (0·06–0·17)	0	0 (0–0)
Parainfluenza 3	0·58 (0·37–1·01)	1·33 (0·96–2·28)	0·62 (0·11–1·3)	0·18 (0·05–0·38)	0·36 (0·1–0·84)	0·19 (0·07–0·8)
Respiratory syncytial virus	5·39 (4·84–6·31)	7·32 (6·45–8·56)	4·9 (4·28–5·79)	2·63 (1·94–3·32)	6·06 (5·25–7·23)	7·24 (4·88–9·3)
Rubella	0·12 (0·07–0·23)	0	0	0·43 (0·25–0·82)	0	0
Other or none§	60·42 (57·69–62·87)	46·18 (41·95–49·84)	72·18 (67·35–76·43)	65·18 (61·89–68·16)	58·26 (53·75–62)	61·95 (55·16–67·73)

Data are means with 95% credible intervals.

* Aggregated incidence was calculated by weighting site-specific incidence estimates by average registered livebirths per month.

† Rates are based on the estimated pathogen prevalence among possible serious bacterial infection episodes, derived from the partially latent class model used to assess 5253 episodes identified among 63 114 registered livebirths.

‡ Includes all bacteria that grew on blood culture but did not have an associated assay on the ANISA molecular diagnostic panel, and which were estimated indirectly by calculating the product of the number of blood culture isolates in this class and the average estimated causal proportion attributed to pathogens with multiple tests that included blood culture.

§ Includes any episode of possible serious bacterial infection not attributed by the partially latent class model to one of the ANISA pathogen classes.

The overall incidence of viral infections was 10·1 (95% CrI 9·4–11·6) per 1000 livebirths (range across sites 5·5–15·7 per 1000 livebirths, table 5). The incidence of respiratory syncytial virus was greater than 2·5 per 1000 livebirths in all sites, peaking in Sylhet (7·3 infections per 1000 livebirths). For all other viruses, the incidence was 1·1 per 1000 livebirths or lower, with site-specific variations (table 5).

Overall, among 71 361 livebirths, 3061 (4%) babies died before age 60 days (range 11 per 1000 livebirths in Vellore to 57 per 1000 livebirths in Matiari; overall mortality 42·9 per 1000 livebirths). 1377 (45%) of these 3061 deaths occurred among non-registered babies (1284 within 7 days and 93 after 7 days) and 1684 (55%) among registered babies. Among 1377 babies who were not visited by a CHW before death, 941 (68%) died within 24 h of birth (median age 6·1 h, appendix). Mortality among registered babies was 26·7 deaths per 1000 livebirths, which was 7·4 (95% CrI 6·7–8·1) times higher among babies with pSBI than among healthy babies (125·0 *vs* 16·9 per 1000 livebirths). Among the 1684 registered babies who died, 689 (41%) were assessed by a physician before death and pSBI was confirmed. Samples were collected from 349 (49%) of these confirmed episodes in the 7 days before death (appendix). Among 102 babies with confirmed episodes who died within 24 h of birth, 37 (36%) had samples collected.

**Figure 3:**
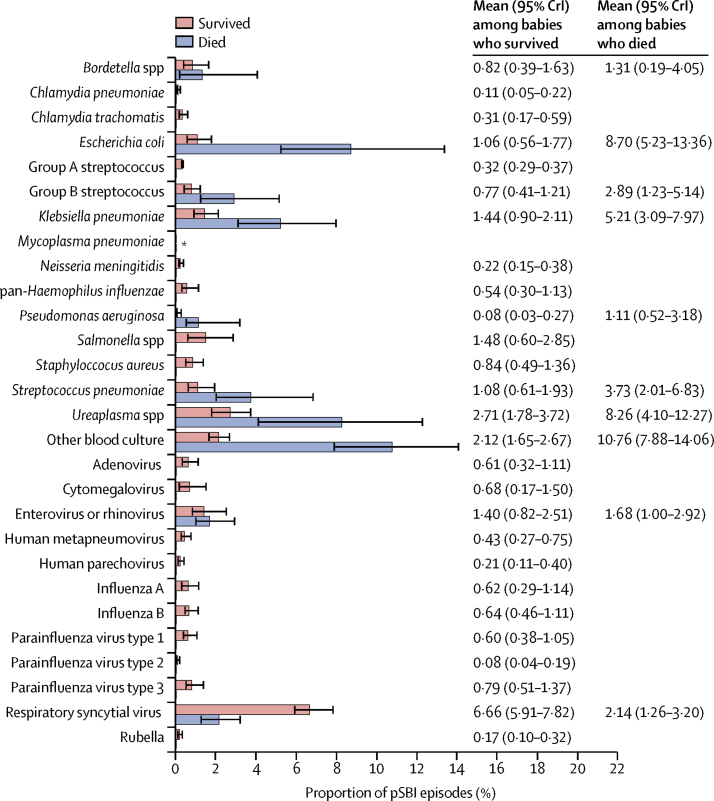
Attributed proportions of causal pathogens tested during possible serious bacterial infection episodes among babies who died and survived, estimated in a partially latent class model CrI=credible interval. *None detected.

Causal pathogen attributions were made in substantially more babies with pSBI episodes who died than among those who survived (46%, 95% CrI 37–56 *vs* 27%, 24–30). Of the deaths among babies with pSBI episodes with attributed pathogens, 92% were due to bacteria, led by *E coli* (causal proportion 8·7%, 95% CrI 5·2–13·4) and *Ureaplasma* spp (8·3%, 4·1–12·3; figure 3). Other pathogens that were over-represented among babies with pSBI who died compared with among those who survived were *K pneumoniae, S pneumoniae*, group B streptococcus, and *Pseudomonas aeruginosa* (figure 3). In contrast, the proportion attributed to respiratory syncytial virus was greater among babies who died than among those who survived (2·1%, 95% CrI 1·3–3·2 *vs* 6·7%, 5·9–7·8).

Given these differences in pathogen distributions between babies who died and those who survived and the low capture of samples within 7 days of life (11·6% of deaths), we did a sensitivity analysis to ascertain the potential effect on the overall distribution of causal organisms. Specifically, we assumed that the pathogens among missed deaths were distributed equivalently to those among deaths with laboratory results in the most extreme scenario (100% of deaths fulfilling the pSBI definition). The overall pathogen prevalence distribution in ANISA did not change markedly, with the pathogen proportion attributed to *Ureaplasma* spp*, E coli*, and *K pneumoniae* being increased to about 3·0–4·0%, and those attributed to respiratory syncytial virus decreased from 6·5% to 5·3% (appendix).

## Discussion

With neonatal deaths fast becoming predominant among deaths in children younger than 5 years, we aimed with the ANISA study to use state-of-the-art microbiology techniques and computational modelling in one of the largest community-based studies of its kind to bridge major knowledge gaps and improve estimates of incidence of neonatal infections and their causes in south Asia, where 36% of all neonatal deaths occur. Although causal attribution among babies with pSBI in our cohort of over 63 000 livebirths was low (28%) because of the highly sensitive clinical algorithm we used, we found that bacterial causes predominated over viral causes. Even more importantly, ANISA captured data on pathogens associated with pSBI deaths, indicating that a cause could be attributed in 46% of deaths, among which most (92%) were bacterial.

The incidence of culture-confirmed bacterial pSBI episodes was 1·6 per 1000 livebirths, which was lower than previously reported incidence in south Asia (3·0 per 1000 livebirths in Mirzapur, Bangladesh,^12^ and 6·7 per 1000 livebirths in Odisha, India^13^). A study in Africa reported incidence of 5·5 per 1000 livebirths for bacterial infections among neonates.^23^ The low frequency of culture-confirmed pSBI episodes in ANISA might have stemmed partly from efforts to minimise inclusion of isolates that were not clinically relevant, strategies to minimise contamination of samples, and expert review of positive cultures. Use of the molecular assays and modelling to attribute causes, however, indicated a higher proportion of bacterial causes than reported in previous studies.

Among bacterial causes, *Ureaplasma* spp predominated, particularly during the first 3 days of life and among babies with pSBI who died. The role of this atypical bacterium as a cause of neonatal morbidity and mortality is not well understood because it does not grow under conventional culture methods. Previous associations of *Ureaplasma* spp with adverse outcomes in mothers (chorioamnionitis and extreme preterm delivery) and neonates (prematurity, sepsis or meningitis, and pulmonary and cerebral morbidity) have been documented, as have pSBI episodes in neonates due to *U parvum* and *U urealyticum*.^6^, ^24^ Common empirical treatment with β-lactams or cephalosporins is not effective against *Ureaplasma* spp due to the lack of a cell wall.^25^ Future studies using various methods are needed to characterise the pathogenic role of *Ureaplasma* spp in babies and guide treatment policies.

Over half of bacteria identified as causing pSBI were Gram negative, particularly *Klebsiella* spp and *E coli*. These bacteria ranked among the top pathogens resulting in death among babies with pSBI episodes and were present in all study sites. Blood culture analysis revealed higher incidence of Gram-negative organisms among hospital-born babies than among community-born babies. These findings are consistent with those in previous studies from south Asia.^8^, ^13^ The prevalence of Gram-negative organisms in the environment and on the body surface of neonates in south Asia might be the source of these infections.^26^, ^27^

In concordance with previous data from south Asia, the incidence of pSBI episodes caused by group B streptococcus was lower than that reported in Africa.^28^, ^29^ Attribution was, however, more than 3·7 times more likely among babies with pSBI who died than among survivors, and 1·8 times more likely among babies with early-onset illness than among those with late-onset illness. These findings suggest that the burden of group B streptococcus might be underestimated because of the challenges in capturing samples after early death. A study done in Bangladesh at the same site as ANISA showed that mortality was increased by 6·6 times among neonates with group B streptococcus colonisation of the umbilical cord stump.^30^ Further investigations of early deaths and a maternal vaccine probe study will be crucial to understanding the true burden of group B streptococcus among babies in south Asia.

We collected 349 samples from babies who died, which exceeds the number of samples obtained from babies after pSBI-associated deaths in previous studies, yet still represents only a small proportion of all deaths in the ANISA catchment areas, despite intensive efforts. This notable limitation stemmed from the high frequency of early deaths. 68% of deaths among non-registered babies occurred within 24 h of birth (median age 6·1 h), among whom over a quarter died in the first hour of life (appendix). The challenge of reaching babies before death also translates into challenges for the treatment and prevention of illnesses in at-risk babies. Among those who had pSBI episodes and died, the causative bacteria were diverse (>25 species within 19 genera), complicating development of empirical treatment and prevention strategies. Furthermore, antibiotic therapy might be given too late to be effective, particularly for births outside health facilities. Maternal immunisation has the potential to protect fetuses and neonates from infection, but maternal vaccines are not available for any of the leading organisms associated with pSBI deaths. Interventions such as use of a clean delivery kit at birth might prevent some early deaths due to infection.

The predominant role of respiratory syncytial virus (5·4 per 1000 livebirths) among viral causes of pSBI in the first 2 months of life is consistent with previous studies in neonates in Bangladesh and Nepal.^14^, ^31^ Among babies who had pSBI and died in this study, respiratory syncytial virus ranked sixth. Respiratory syncytial virus further stresses under-resourced health systems where the numbers of hospital beds are small and patients need continuous care for common sequelae, such as recurrent wheeze and asthma. The contribution in this study of respiratory syncytial virus to pSBI, even in the first days of life, suggests a specific opportunity to intervene and prevent deaths and morbidity through maternal immunisation, administration of monoclonal antibodies to neonates, or both, which are looking likely to become available options.^32^, ^33^

Our findings should be considered within the context of some limitations. First, despite using highly sensitive diagnostic methods, we could not identify causes in most (72%) pSBI episodes, and more than half (54%) of fatal pSBI episodes. The reasons for these outcomes are multifactorial. First, a subset of episodes could have had causative agents beyond the 28 prioritised by the expert panel. The unexpected finding of *Ureaplasma* spp as leading causes indicates that organisms that are not generally judged to be the most common might have important roles. Genomic analyses have shown that many bacterial species can only be detected by culture-independent approaches.^34^, ^35^ With advances in metagenomics, the biobank of the ANISA study will provide an opportunity to probe for additional organisms. Second, ANISA only captured blood and respiratory samples. Although cerebrospinal fluid collection was attempted, very few samples were obtained. We did not try to collect samples from other sterile sites, such as lung aspirates or urine, or peripheral sites other than the nasopharynx and oropharynx. Third, collection of blood and respiratory samples from healthy babies in the first days of life was challenging due to difficulties in early birth capture and community acceptability. Thus, samples from healthy babies were skewed towards older babies who might have had more time to acquire organisms from the environment. Overestimation of background presence of potential pSBI organisms would, therefore, have underestimated causal attribution. Fourth, we used WHO's Integrated Management of Childhood Illness algorithm to define pSBI, but this approach emphasises sensitivity at the cost of specificity.^36^, ^37^ The standard clinical signs of pSBI also overlap with those of intrapartum-related events and other non-infectious causes of neonatal mortality, making it unlikely that all pSBI episodes are of infectious origin. Our findings lend support to the prediction of Mulholland and colleagues^38^ that only a limited proportion of babies with pSBI actually have illnesses of bacterial origin. Moreover, poor specificity could explain the results of other studies in which standard and simplified antibiotic regimens (ranging from 14 to two injections and first-line oral antibiotics) were equally effective.^39^, ^40^, ^41^ We found that 83% of the true bacterial isolates from pSBI were sensitive to first-line antibiotics, which suggests that widespread antimicrobial resistance is not yet deeply rooted in the community.

ANISA has opened several avenues for further exploration that might uncover new strategies for improving survival among babies and achieving Sustainable Development Goal 3. These include application of the latest metagenomic approaches for improved characterisation of causal organisms in samples; the role of *Ureaplasma* spp, for which studies should be designed to elucidate management policies; assessment of maternal immunisation against respiratory syncytial virus infection; identification of ways to differentiate infectious from non-infectious pSBI (eg, with biomarkers or distinct clinical signs, metagenomics approaches, and minimally invasive tissue sampling^42^); and investigation of factors unrelated to infection that cause pSBI. Low antibiotic resistance across south Asian communities provides an incentive to limit unnecessary antibiotic use to prevent emergence of antimicrobial resistance.
