# Supplementary material for: Causes and incidence of community-acquired serious infections among young children in south Asia (ANISA): an observational cohort study
Source: Lancet. 2018 Jul 14;392(10142):145–59. doi: 10.1016/S0140-6736(18)31127-9 (PMC6053599; doi:10.1016/S0140-6736(18)31127-9)
Supplement: Supplementary appendix [file mmc1.pdf]

THE LANCET

Supplementary appendix

This appendix formed part of the original submission and has been peer reviewed. We post it as supplied by the authors.

Supplement to: Saha SK, Schrag SJ, El Arifeen S, et al. Causes and incidence of community-acquired serious infections among young children in south Asia (ANISA): an observational cohort study. *Lancet* 2018; published online July 6. http://dx.doi.org/10.1016/S0140-6736(18)31127-9.

SM 1. Aetiologic attribution: Summary of the partial latent class model used in Aetiology of Neonatal Infections in South Asia (ANISA) Study

1. The basic pLCM (partially-Latent Class Model):

ANISA statistical methodology was an extension of the basic partially-Latent Class Model (pLCM) developed by Wu et al for the Pneumonia Etiology Research for Child Health (PERCH) to estimate the proportion of pneumonia infections attributed to specific pathogens.[1,2] The structure of the basic pLCM used in ANISA can be summarized as below.

Suppose there are K targeted pathogens (as on the TAC cards or isolated by blood culture in ANISA) each with one diagnostic test $T_k, k = 1, \dots K$ that produces binary (positive vs negative, or 1 vs. 0) test result y_{ik} for case $i, i = 1, \dots, N$. We add one extra class (referred to throughout as 'Other/None') for other pathogenic or non-pathogenic causes and code it as class $(K + 1)$. If we assume each individual case has only one etiology cause from the $K + 1$ classes, then the population of cases can be considered as a mixture of subpopulations with etiology $k = 1, \dots, K, K + 1$. Let Z_i be the true (unobserved) etiology of case $i, i = 1, \dots, N$, then the objective of the model is to estimate probability $\pi_k = P(Z_i = k), k = 1,2, \dots, K, K + 1$, using the observed binary test results $\{y_{ik}, k = 1, \dots, K; i = 1, \dots, N\}$. Here $0 \le \pi_k \le 1$ and $\sum_{k=1}^{K+1} \pi_k = 1$. We refer to π_k as pathogen proportion throughout.

pLCM expresses the probability of observing $\{y_{ik}\}$ through a linear mixture model with $\{\pi_k\}$ as the mixing coefficients. By applying the regular conditional independence assumption for such linear mixture class models with unknown (latent) classes, and a further assumption that the probability of test T_k to produce positive test result depends only on whether pathogen k is the true etiology of the tested case, the linear mixture can be simplified as:

$$f(y_{ik}, k = 1, \dots, K; i = 1, \dots, N) = \prod_{i=1}^{n} \left(\sum_{k=1}^{K} \pi_k \theta_k^{y_{ik}} (1 - \theta_k)^{1-y_{ik}} \prod_{j \neq k} \delta_j^{y_{ij}} \left(1 - \delta_j\right)^{1-y_{ij}} + \pi_{K+1} \prod_j \delta_j^{y_{ij}} \left(1 - \delta_j\right)^{1-y_{ij}}\right) \quad [1]$$

Here parameters $\theta_k = f(y_{ik} = 1 \mid true\ etiology = k)$ and $\delta_k = f(y_{ik} = 1 \mid true\ etiology\ \neq k)$ are called the True Positive Rate (TPR) and False Positive Rate (FPR) respectively for test $T_k, k = 1, \dots K$.

The parameters in Equation 1 can be estimated under a Bayesian analysis framework using conjugate priors for the parameters, for example, (K+1)-class Dirichlet distributions for the pathogen proportions and Beta distributions for the TPRs and FPRs. With minor modifications, the basic pLCM model can be extended to situations where multiple pathogen-specific tests are performed, including blood culture. Additional pathogen classes could be created to account for pathogen co-infections. [3]

The performance of pLCM was evaluated through simulation studies.[4,5] Knoll et al demonstrated that pLCM outperforms the regular etiology fraction method based on population attributable risk (PAR). Further, they showed that with a large sample of healthy controls (serving as a negative gold standard), an adequate number of cases with confirmed pathogen infection (such as through blood culture isolation), and prior knowledge of the TPRs associated with diagnostic tests used for a subset of pathogens, the basic pLCM will usually produce reliable pathogen proportion estimates. More extensive simulation experiments by Shang et al showed that even with non-informative priors, pLCM can still estimate pathogen proportions reliably, especially for the class of "Others/None", if at least a few pathogens are tested by more than one laboratory test. Our simulation experiments also revealed a few situations where pLCM might perform less well, notably when pathogens only had a single laboratory test performed, true pathogen proportions were very low and either FPR values were high or TPR and FPR were close in value. In ANISA we developed mitigation strategies (see Section 3) to avoid inclusion of such pathogens in the pLCM, and where mitigation was not possible, we excluded such pathogens by combining them intothe "Other/None" class.

2. An extension of pLCM with covariate dependent pathogen proportions and false positive rates

The TAC tests employed in ANISA were developed by extensive testing of the target pathogens and nearest neighbors to ensure high laboratory specificity. Hence a positive test result almost certainly indicated that the pathogen was present in the collected specimen. Since we assume a single etiology cause for each case, positives for non-etiological pathogens indicate pathogen carriage. Because pathogen carriage rates likely change over many covariates such as location (study site), season (enrollment date) and age, false positive rates in pLCM should vary similarly. However, the assignment of pathogen classes as the cause of a pSBI episode is established by comparing the true and false positive rates. If false positive rates vary by covariates, an identical set of test results may have different etiologic meaning at different covariate values. Consequently, pathogen proportions cannot be determined by test results alone but should also vary by covariates. Thus we extended the basic pLCM to the following model:

$$f(\tilde{Y}_1,\cdots,\tilde{Y}_n) = \prod_{i=1}^{n}(\sum_{k=1}^{K}\pi_k^{x_i}\theta_k^{y_{ik}}(1-\theta_k)^{1-y_{ik}}\prod_{l\neq k}(\delta_l^{x_i})^{y_{il}}\left(1-\delta_l^{x_i}\right)^{1-y_{il}} + \pi_{K+1}^{x_i}\prod_l(\delta_l^{x_i})^{y_{il}}\left(1-\delta_l^{x_i}\right)^{1-y_{il}}) \quad [2]$$

Here $(x_1,\dots,x_n)$ are the observed values of the covariates for the *n* cases. $\widetilde{\Pi}^x = (\pi_1^x,\cdots,\pi_K^x,\pi_{K+1}^x)$ and $\widetilde{\Delta}^x = (\delta_1^x,\cdots,\delta_K^x)$ are the pathogen proportion distributions and false positive rates for any $x \in \mathcal{X}$. Notice that we hold the true positive rates $\Theta = (\theta_1,\dots,\theta_K)$ constant across covariate levels because we assume that infection by a pathogen implies presence of the pathogen in the respiratory or blood samples from cases.

Allowing false positive rates to vary by covariates is not only necessary to address known variation in pathogen carriage, but also alleviates violations of the conditional independence assumption in pLCM. This is because covariates are often confounders for co-carriage of some pathogens. Through adjusting the confounding effect, dependence between test results for co-carried pathogens may weaken, both locally and globally.

A Bayesian Kernel Model approach was developed to estimate parameters in the extended model as expressed by Equation 2. For each data point x in the domain of the covariates, we assume pathogen proportion $\widetilde{\Pi}^x = (\pi_1^x, \cdots, \pi_K^x, \pi_{K+1}^x)$ to have a Dirchlet prior with parameters$(e_1^{x0}, \cdots, e_K^{x0}, e_{K+1}^{x0})$, and the false positive rates $\delta_k^x, k = 1, \dots, K$ to have Beta priors with parameters $\left(c_k^{x0}, d_k^{x0}\right), k = 1, \dots, K$. We also assume the posterior distributions of the parameters can be approximated by the same type of distributions and thus use them as the sampling distributions in the next iteration of the Gibbs Sampler. The parameters of the sampling distributions are updated by the following equations:

$$e_k^x = e_k^{x0} + \sum_{i=1}^N z_{ik} \times d(x, x_i), k = 1, \dots, K, K+1 \qquad [3]$$

$$c_k^x = c_k^{x0} + \sum_{i=1}^N y_{ik} \times z_{i(K+1)} \times d(x, x_i), k = 1, \dots, K \qquad [4a]$$

$$d_k^x = d_k^{x0} + \sum_{i=1}^N (1 - y_{ik}) \times z_{i(K+1)} \times d(x, x_i), k = 1, \dots, K \qquad [4b]$$

Here $Z_i = \left(z_{i1}, z_{i2}, \dots, z_{iK}, z_{i(K+1)}\right)$ are the imputed pathogen (latent) classes for the etiology of case *i* from the previous iteration of the Gibbs sampler. z_{ik} takes value 0 or 1 only, and $\sum_{k=1}^{K+1} z_{ik} = 1$. The quantity $d(x, x_i)$ measures the contribution of case *i* at x_i to data point x, such that $0 \leq d(\mathrm{x}, x_i) \leq 1$, $\max_x d(x, x_i) = d(x_i, x_i) = 1$, and $d(x, x_i)$ decreases as the distance between x and x_i increases. For discrete covariates, $d(x, x_i)$ takes value 1 or 0 depending whether x_i and x share the same covariate values. For continuous covariates

$$d(x, x_i) = C \times K(x \mid x_i, h) \qquad [5]$$

Here $K(x \mid x_i, h)$ is a Gaussian density function with mean at $x_i$and standard deviation h. C is a constant to make sure that $d(x_i, x_i) = 1$.

The prior distributions of parameters are constructed by adding (K+1) pseudo cases to the study population (one pseudo case per pathogen class). Each pseudo case has equal probability to be positive or negative for each of the laboratory tests. Hence, the prior distribution for the true positive rates, which are invariant by covariates,

are the Jeffery non-informative prior for binary events, or Beta(0.5, 0.5). When covariates are considered, each pseudo case will be considered as a probability density function uniformly distributed on the domain $\mathcal{X}$ of the covariates. Its overall contribution to a data point $x \in \mathcal{X}$ is then:

$$d(x) = \frac{1}{|\mathcal{X}|}\int_{t \in \mathcal{X}} d(x,t)dt \qquad [6]$$

Here we assume the domain of the covariates is finite and $|\mathcal{X}|$ is its volume (area). Hence, the prior for the pathogen distribution at data point x will be $Dirichlet(d(x), d(x), \ldots, d(x))$. The overall contribution of the prior distributions to our analyses (= (K+1) / N) is very small if the number of targeted pathogen K is much smaller than the number of cases N.

The prior distribution for false positive rates is constructed from the control data, by calculating contributions of all control data points to $x \in \mathcal{X}$, using the same function $d(x,y)$. For a particular test T, we add the contributions from controls with positive and negative results as the Beta parameters for the false positive rate.

The smoothing parameter h in Equation [5] controls the amount of local smoothing. It can be decomposed into two components: $h = \sqrt{h_c^2 + h_d^2}$. Here h_d controls the smoothness of the estimated probability density of the covariates. We use the "rule of thumb" in the density estimation literature to set h_d . For example, if N is the sample size of cases and there are two continuous covariates, then $h_d = N^{-1/6}$ after the covariates are scaled with mean 0.0 and variance 1.0. h_c is used to define the neighborhood of data points. We applied knowledge of the epidemiology of the disease under evaluation to narrow the range of the parameters.

3. Implementation details of the extended pLCM model

Two strategies were implemented to mitigate inclusion of pathogens with characteristics that might result in unreliable model performance based on simulation experiments (see Section 1). First, we prescreened pathogen lists within each study site and each case outcome status (died vs. survived) using a stepwise procedure that excluded pathogens with very few positive TAC tests results. The second strategy flagged remaining pathogens with: a) high false positive rates among controls; b) lower odds ratio between cases and controls; and c) significant and substantial pairwise correlations of tests results among controls. The effects of the covariates on flagged pathogens were further examined through stratifications and/or regression models. If the covariates did not reduce the flagged features at least locally, then flagged pathogens were considered for exclusion from the model (no pathogens in ANISA fell in this category). If the flagged features disappeared in some local regions of covariates, but remained in others, then the pathogen was kept in the model, but local estimations in the troubled regions should be interpreted with caution. If a pathogen was not selected in any of the strata, the pathogen effectively was captured by the "Other/None" class. If a pathogen was included in some strata, but not others, then the pathogen proportion was set to zero in the strata where the pathogen was excluded from the target list; because this occurred only for pathogens with very few or no positive test results, setting to zero was the best approximation.

In ANISA, non-informative priors were used for all parameters. The contribution of the priors was equivalent to adding one pseudo case per pathogen class into the case population, or 28 pseudo cases to a population of approximately 5,300. Thus, the contribution of priors was so small that our results can be considered as data driven, rather than prior distribution driven. Additionally, in ANISA we set lower limits for TAC test true positive rates: 40% for respiratory TAC and 20% for Blood TAC tests. We did not set a lower limit for blood culture true positive rates.

The neighborhood smoothing parameter h_c was chosen based on the known epidemiology of sepsis: 3 days for age (with a range of 60 days) and 3 months for enrollment time (with an average range of 24 months). With this choice of smoothing parameter, the average local sample size, defined as $N_{x,h} = \sum_{i=1}^{N} d(x, x_i)$, was approximately 200 cases in a study site with 1,000 enrolled cases (the average site of the non-India ANISA sites). In other words, instead of using 1,000 cases to estimate covariate independent pathogen proportions and false positive rates, the extended pLCM uses an average of 200 cases to estimate parameters that vary by the covariates. The local sample size of 200 appeared capable of capturing major seasonal and age trends, without creating unstable local random fluctuations.

When updating distributions for false positive rates in Equation [4a] and [4b], only cases assigned to class (K+1), or the class of "Other/None", were included in order to minimize a concern that infection by a pathogen might change carriage rates of other pathogens.

After a 50,000 iteration burn-in period we ran the ANISA pLCM for 150,000 iterations. Posterior means and the corresponding 95·0% credible intervals of model parameters were then generated from the iterations, either globally or stratified by covariates, or even individually for cases.

, posterior means and the corresponding 95·0% credible intervals of model parameters were then generated from the corresponding samples, either globally or stratified by covariates, or even individually for cases. Continuous two-dimensional (for age and enrollment) heat maps, as well as one-dimensional marginal or conditional curves, can also be constructed to visually reveal age and seasonal patterns of etiology proportions for individual pathogens.

The proportions of pathogens that were isolated by blood culture but not on the target list of TAC cards could not be estimated directly by the pLCM model. We combined them into a pathogen class called "Other Blood Culture". The proportion of episodes attributed to this combined class was estimated indirectly by calculating the product of the number of blood culture isolates in this class and the average estimated proportion attributed to pathogens with multiple tests that included blood culture and that yielded at least one isolate.

Because the primary output from the model is pathogen proportion, incidence rates (per 1000 live births), were calculated by the product of the total cases and the pathogen-specific proportions divided by total registered live births. When data across sites were aggregated, site specific estimates were weighted by each site's average monthly cases since sites had different catchment sizes and enrolled for different periods of time

To generate the 150,000 iterations of Gibbs sampler, a total computation time of 80-90 hours was required (2 seconds per iteration). The ANISA computation program was written in R and can be shared upon request.

References

1. Wu, Z., Deloria-Knoll, M., Hammitt, L.L., & Zeger, S.L., Partially latent class models for case-control studies for childhood pneumonia aetiology. Journal of the Royal Statistical Society: Series C (Applied Statistics), 2016. 65(1): p. 97-114.
2. O'Brien KL, Baggett HC, Brooks WA, Feikin DR, Hammitt LL, Howie SRC, Deloria Knoll M, Kotloff KL, Levine OS, Madhi SA, Murdoch DR, Scott JAG, Thea DM, Zeger SL. Introduction to the Epidemiologic Considerations, Analytic Methods, and Foundational Results From the Pneumonia Etiology Research for Child Health Study. Clin Infect Dis. 2017 Jun 15;64(suppl_3):S179-S184. doi: 10.1093/cid/cix142
3. Z. Wu, M. Deloria-Knoll, S. Zeger. *Nested Partially-Latent Class Models for Dependent Binary Data; Estimating Disease Etiology*. Biostatistics **2016 (16), 00, p. 1-14**.
4. Deloria Knoll, M., et al., *Bayesian Estimation of Pneumonia Etiology: Epidemiologic Considerations and Applications to the Pneumonia Etiology Research for Child Health Study.* Clin Infect Dis, 2017. 64(suppl_3): p. S213-s227.
5. Shang, N, Arvay, ML, Liu, A, Mullany, LC, Schrag, SJ. Evaluation of a Bayesian partial latent class method for etiologic attribution: application to the Aetiology of Neonatal Infections in South Asia (ANISA) study. Canadian Journal of Infectious Diseases and Medical Microbiology. Submitted.

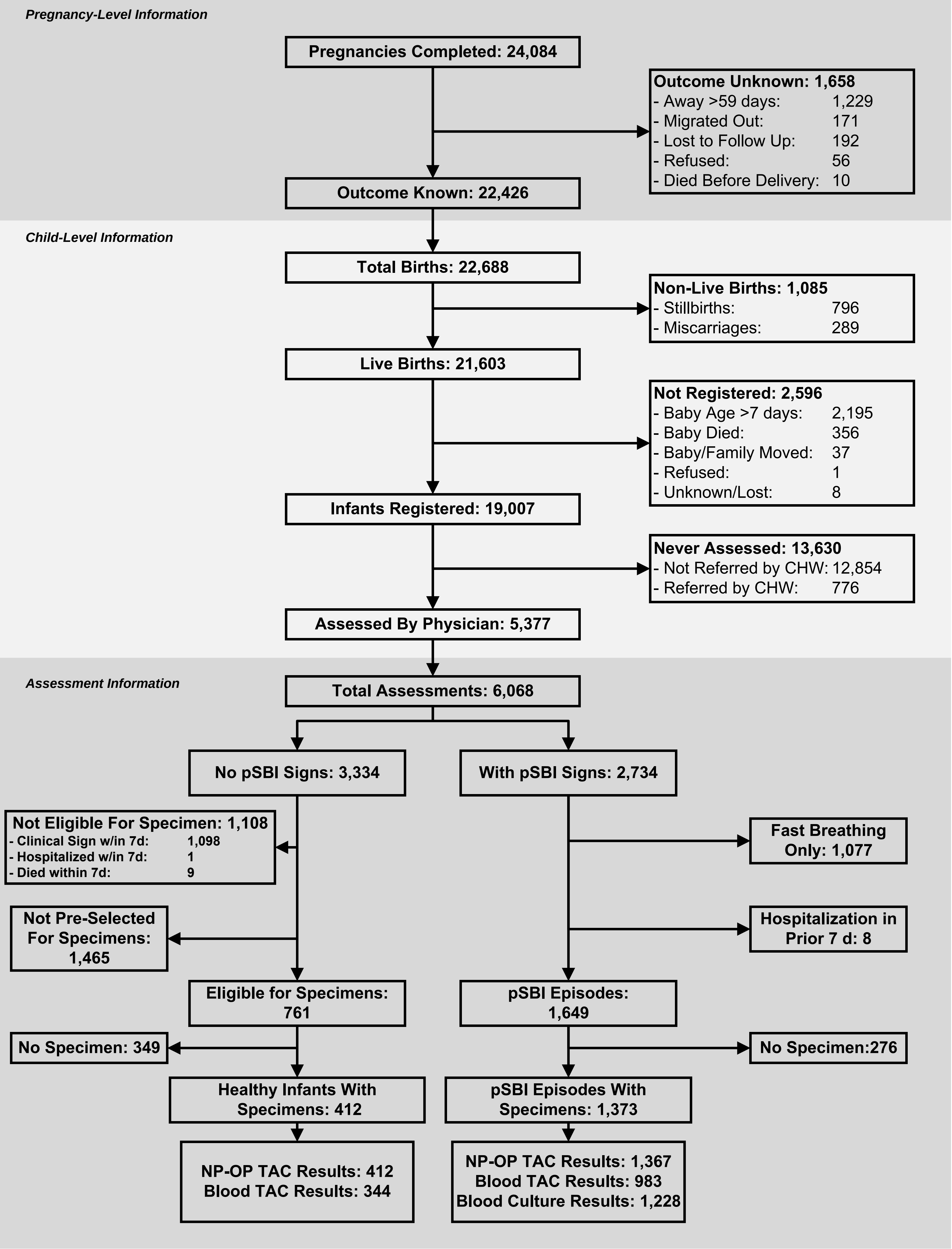

Pregnancy-Level Information
Pregnancies Completed: 24,084
Outcome Unknown: 1,658
- Away >59 days: 1,229
- Migrated Out: 171
- Lost to Follow Up: 192
- Refused: 56
- Died Before Delivery: 10
Outcome Known: 22,426
Child-Level Information
Total Births: 22,688
Non-Live Births: 1,085
- Stillbirths: 796
- Miscarriages: 289
Live Births: 21,603
Not Registered: 2,596
- Baby Age >7 days: 2,195
- Baby Died: 356
- Baby/Family Moved: 37
- Refused: 1
- Unknown/Lost: 8
Infants Registered: 19,007
Never Assessed: 13,630
- Not Referred by CHW: 12,854
- Referred by CHW: 776
Assessed By Physician: 5,377
Assessment Information
Total Assessments: 6,068
No pSBI Signs: 3,334
With pSBI Signs: 2,734
Not Eligible For Specimen: 1,108
- Clinical Sign w/in 7d: 1,098
- Hospitalized w/in 7d: 1
- Died within 7d: 9
Fast Breathing Only: 1,077
Not Pre-Selected For Specimens: 1,465
Hospitalization in Prior 7 d: 8
Eligible for Specimens: 761
pSBI Episodes: 1,649
No Specimen: 349
No Specimen:276
Healthy Infants With Specimens: 412
pSBI Episodes With Specimens: 1,373
NP-OP TAC Results: 412
Blood TAC Results: 344
NP-OP TAC Results: 1,367
Blood TAC Results: 983
Blood Culture Results: 1,228

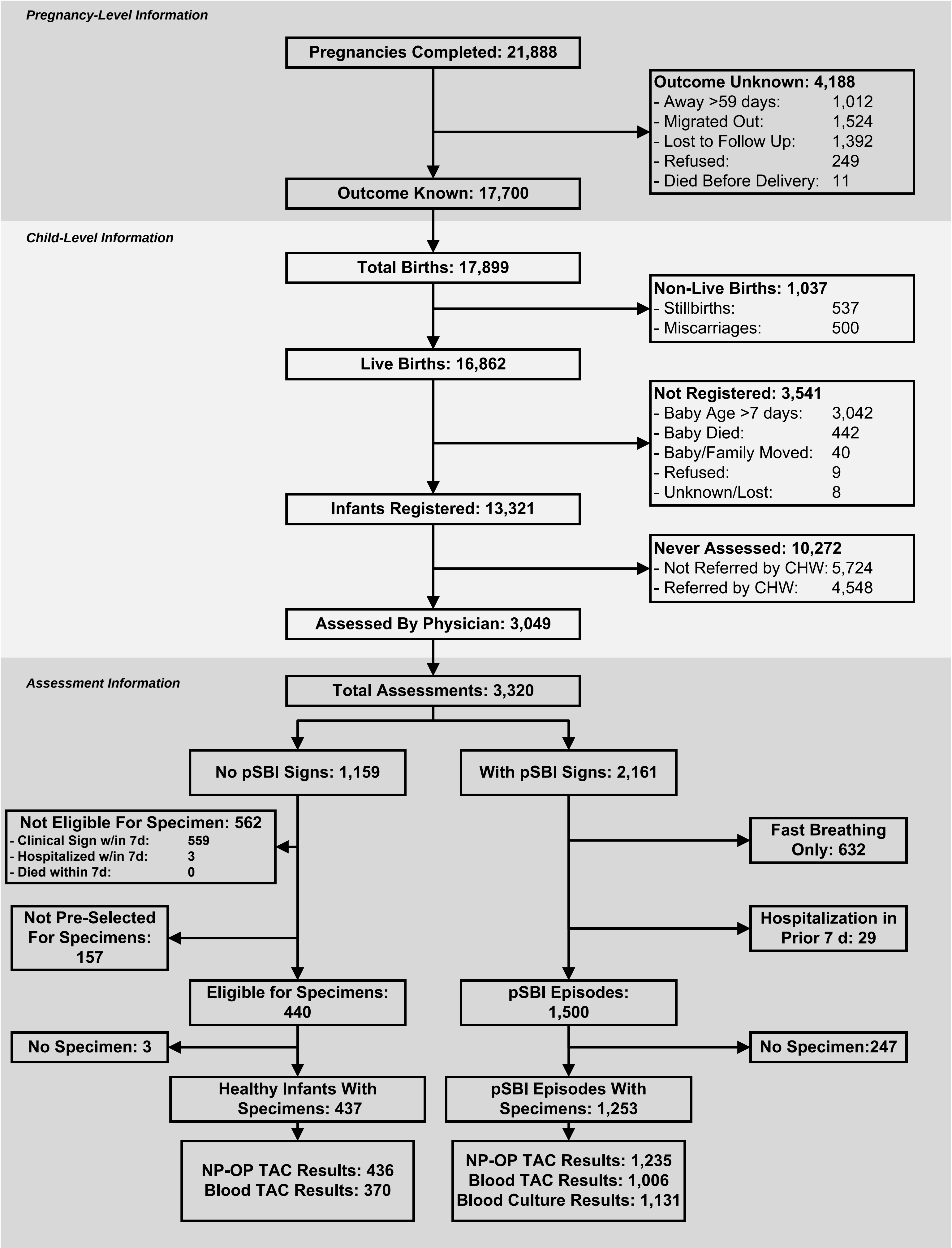
Pregnancy-Level Information
Pregnancies Completed: 21,888
Outcome Unknown: 4,188
- Away >59 days: 1,012
- Migrated Out: 1,524
- Lost to Follow Up: 1,392
- Refused: 249
- Died Before Delivery: 11
Outcome Known: 17,700
Child-Level Information
Total Births: 17,899
Non-Live Births: 1,037
- Stillbirths: 537
- Miscarriages: 500
Live Births: 16,862
Not Registered: 3,541
- Baby Age >7 days: 3,042
- Baby Died: 442
- Baby/Family Moved: 40
- Refused: 9
- Unknown/Lost: 8
Infants Registered: 13,321
Never Assessed: 10,272
- Not Referred by CHW: 5,724
- Referred by CHW: 4,548
Assessed By Physician: 3,049
Assessment Information
Total Assessments: 3,320
No pSBI Signs: 1,159
With pSBI Signs: 2,161
Not Eligible For Specimen: 562
- Clinical Sign w/in 7d: 559
- Hospitalized w/in 7d: 3
- Died within 7d: 0
Fast Breathing Only: 632
Not Pre-Selected For Specimens: 157
Hospitalization in Prior 7 d: 29
Eligible for Specimens: 440
pSBI Episodes: 1,500
No Specimen: 3
No Specimen:247
Healthy Infants With Specimens: 437
pSBI Episodes With Specimens: 1,253
NP-OP TAC Results: 436
Blood TAC Results: 370
NP-OP TAC Results: 1,235
Blood TAC Results: 1,006
Blood Culture Results: 1,131

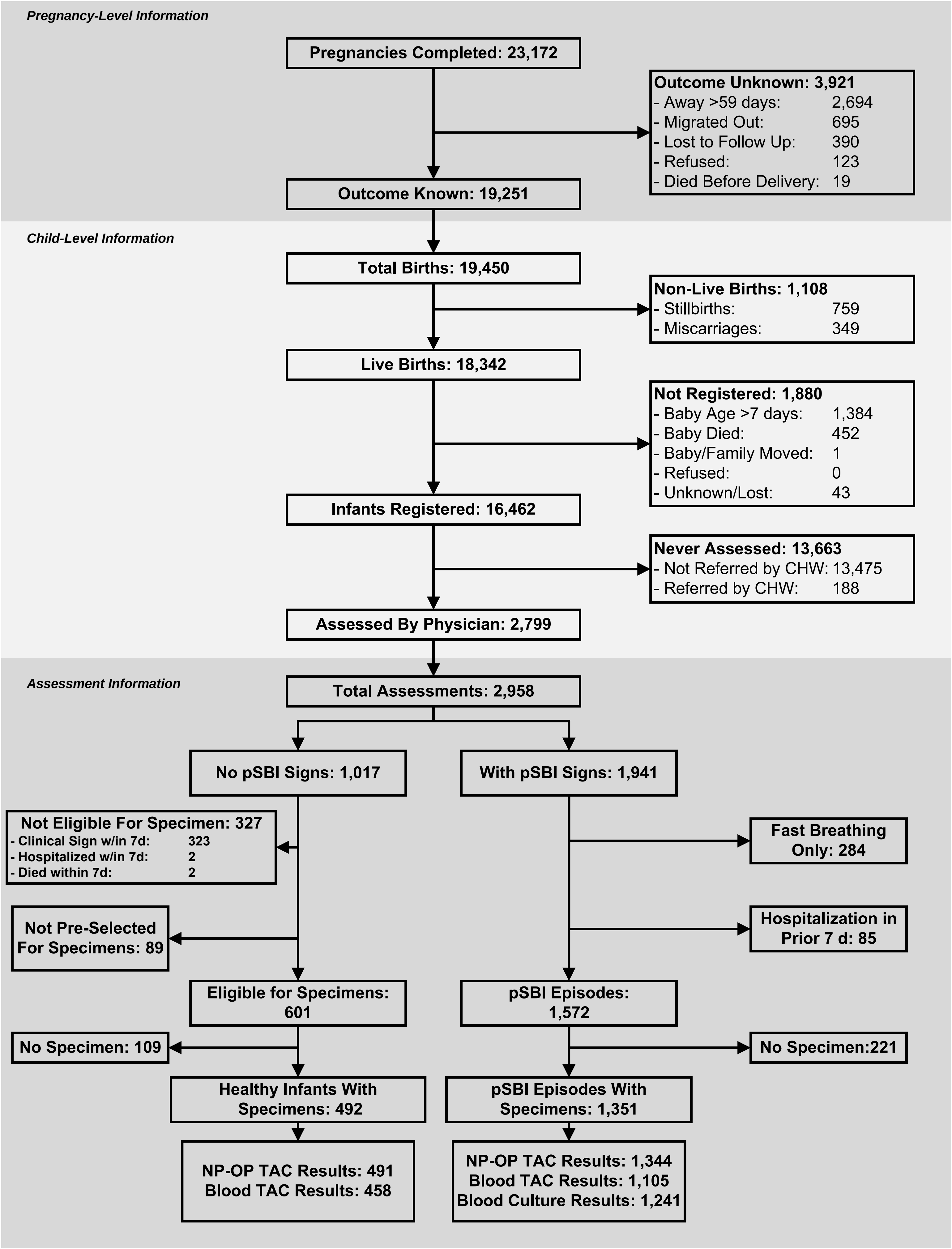
Pregnancy-Level Information
Pregnancies Completed: 23,172
Outcome Unknown: 3,921
- Away >59 days: 2,694
- Migrated Out: 695
- Lost to Follow Up: 390
- Refused: 123
- Died Before Delivery: 19
Outcome Known: 19,251
Child-Level Information
Total Births: 19,450
Non-Live Births: 1,108
- Stillbirths: 759
- Miscarriages: 349
Live Births: 18,342
Not Registered: 1,880
- Baby Age >7 days: 1,384
- Baby Died: 452
- Baby/Family Moved: 1
- Refused: 0
- Unknown/Lost: 43
Infants Registered: 16,462
Never Assessed: 13,663
- Not Referred by CHW: 13,475
- Referred by CHW: 188
Assessed By Physician: 2,799
Assessment Information
Total Assessments: 2,958
No pSBI Signs: 1,017
With pSBI Signs: 1,941
Not Eligible For Specimen: 327
- Clinical Sign w/in 7d: 323
- Hospitalized w/in 7d: 2
- Died within 7d: 2
Fast Breathing Only: 284
Not Pre-Selected For Specimens: 89
Hospitalization in Prior 7 d: 85
Eligible for Specimens: 601
pSBI Episodes: 1,572
No Specimen: 109
No Specimen:221
Healthy Infants With Specimens: 492
pSBI Episodes With Specimens: 1,351
NP-OP TAC Results: 491
Blood TAC Results: 458
NP-OP TAC Results: 1,344
Blood TAC Results: 1,105
Blood Culture Results: 1,241

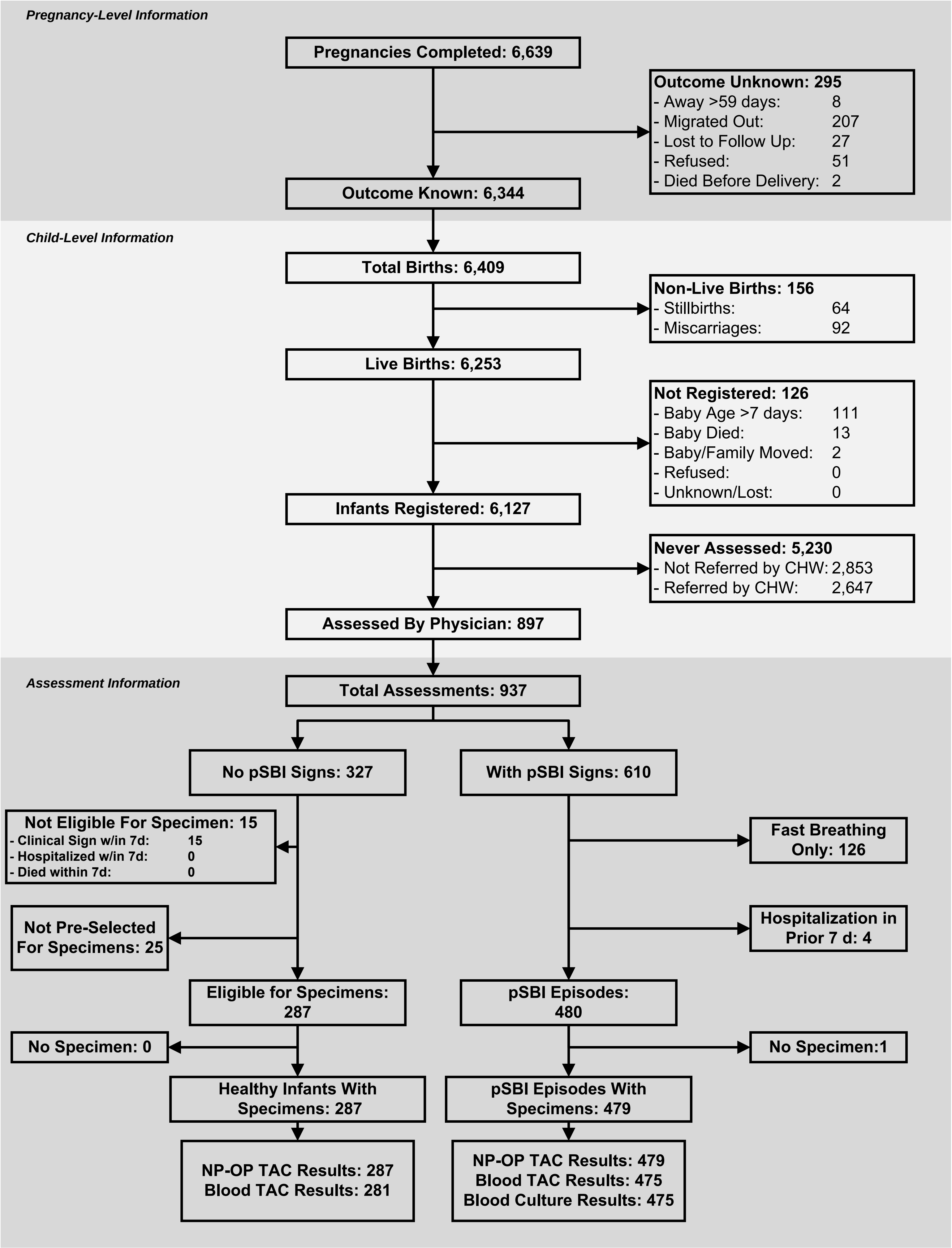

Pregnancy-Level Information
Pregnancies Completed: 6,639
Outcome Unknown: 295
- Away >59 days: 8
- Migrated Out: 207
- Lost to Follow Up: 27
- Refused: 51
- Died Before Delivery: 2
Outcome Known: 6,344
Child-Level Information
Total Births: 6,409
Non-Live Births: 156
- Stillbirths: 64
- Miscarriages: 92
Live Births: 6,253
Not Registered: 126
- Baby Age >7 days: 111
- Baby Died: 13
- Baby/Family Moved: 2
- Refused: 0
- Unknown/Lost: 0
Infants Registered: 6,127
Never Assessed: 5,230
- Not Referred by CHW: 2,853
- Referred by CHW: 2,647
Assessed By Physician: 897
Assessment Information
Total Assessments: 937
No pSBI Signs: 327
With pSBI Signs: 610
Not Eligible For Specimen: 15
- Clinical Sign w/in 7d: 15
- Hospitalized w/in 7d: 0
- Died within 7d: 0
Fast Breathing Only: 126
Not Pre-Selected For Specimens: 25
Hospitalization in Prior 7 d: 4
Eligible for Specimens: 287
pSBI Episodes: 480
No Specimen: 0
No Specimen:1
Healthy Infants With Specimens: 287
pSBI Episodes With Specimens: 479
NP-OP TAC Results: 287
Blood TAC Results: 281
NP-OP TAC Results: 479
Blood TAC Results: 475
Blood Culture Results: 475

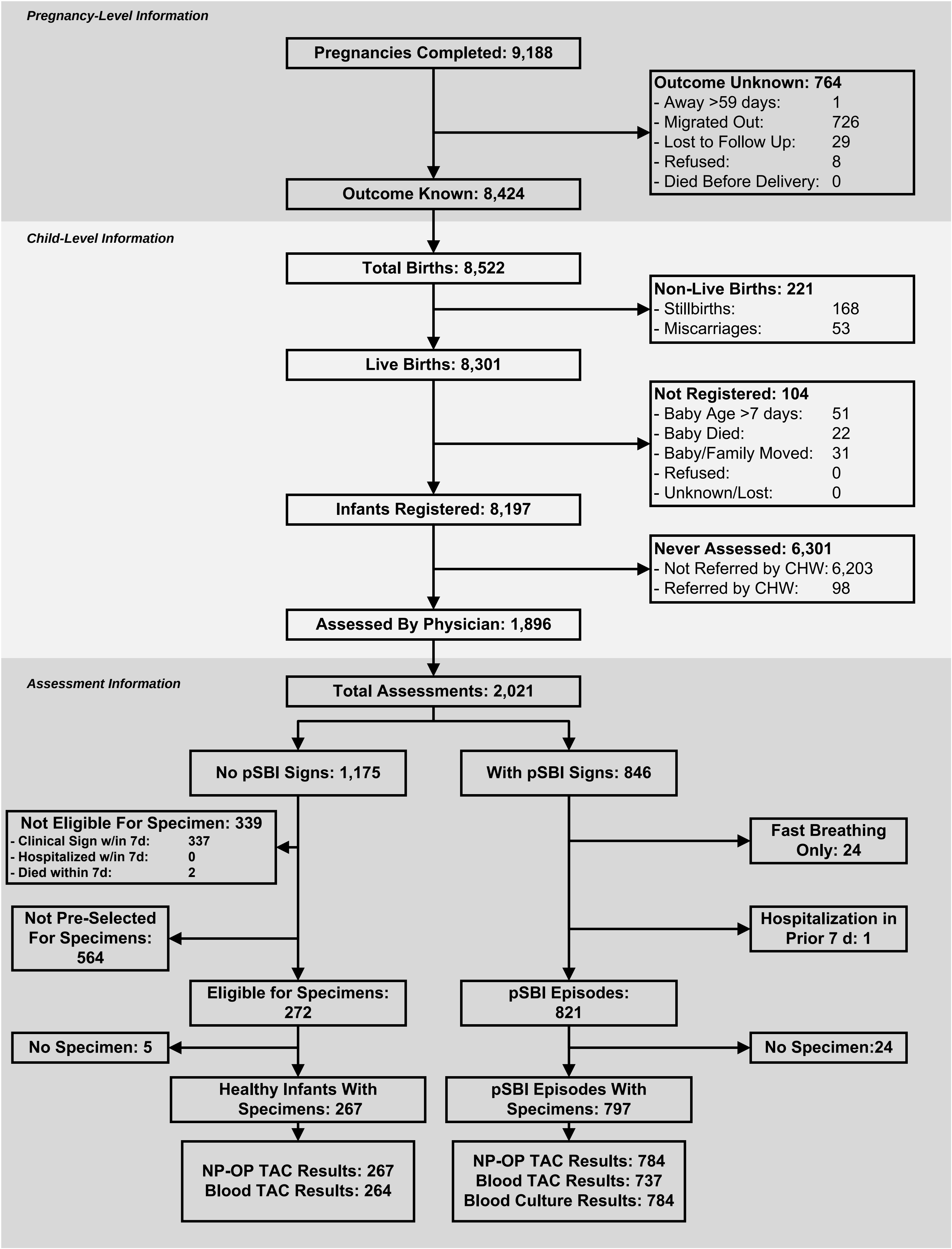

Pregnancy-Level Information
Pregnancies Completed: 9,188
Outcome Unknown: 764
- Away >59 days: 1
- Migrated Out: 726
- Lost to Follow Up: 29
- Refused: 8
- Died Before Delivery: 0
Outcome Known: 8,424
Child-Level Information
Total Births: 8,522
Non-Live Births: 221
- Stillbirths: 168
- Miscarriages: 53
Live Births: 8,301
Not Registered: 104
- Baby Age >7 days: 51
- Baby Died: 22
- Baby/Family Moved: 31
- Refused: 0
- Unknown/Lost: 0
Infants Registered: 8,197
Never Assessed: 6,301
- Not Referred by CHW: 6,203
- Referred by CHW: 98
Assessed By Physician: 1,896
Assessment Information
Total Assessments: 2,021
No pSBI Signs: 1,175
With pSBI Signs: 846
Not Eligible For Specimen: 339
- Clinical Sign w/in 7d: 337
- Hospitalized w/in 7d: 0
- Died within 7d: 2
Fast Breathing Only: 24
Not Pre-Selected For Specimens: 564
Hospitalization in Prior 7 d: 1
Eligible for Specimens: 272
pSBI Episodes: 821
No Specimen: 5
No Specimen:24
Healthy Infants With Specimens: 267
pSBI Episodes With Specimens: 797
NP-OP TAC Results: 267
Blood TAC Results: 264
NP-OP TAC Results: 784
Blood TAC Results: 737
Blood Culture Results: 784

SM 4. Characteristics of healthy infants who provided specimens and their mothers, Aetiology of Neonatal Infections in South Asia (ANISA) Study

Characteristic (% unless otherwise specified)	Sylhet (Bangladesh)	Karachi (Pakistan)	Matiari (Pakistan)	Vellore (India)	Odisha (India)	Total
Characteristic (% unless otherwise specified)						
Maternal	**N=411**	**N=436**	**N=491**	**N=286**	**N=267**	**N=1891**
Age (median (range))	26 (min: 14, max: 53)	26 (min: 14 max: 43)	30 (min: 16, max: 49)	24 (min: 16, max: 44)	24 (min: 14, max: 46)	
First birth	120 (30)	95 (24)	81 (20)	128 (53)	125 (50)	549 (32)
[ζ]Poor nutritional status	62 (15)	34 (8)	40 (8)	20 (7)	20 (8)	176 (9)
*Received full antenatal package	242 (59)	221 (51)	151 (31)	194 (68)	244 (91)	1052 (56)
At least 1 antenatal care visit with a skilled provider	249 (61)	320 (73)	429 (87)	286 (100)	258 (97)	1542 (82)
Birth location						
Health facility	40 (10)	245 (56)	340 (69)	285 (100)	259 (97)	1169 (62)
Home	368 (90)	191 (44)	151 (31)	1 (0)	8 (3)	719 (38)
[ʒ]Skilled birth attendant	53 (13)	259 (59)	339 (69)	285 (100))	246 (92)	1182 (63)
Clean delivery kit	50 (12)	342 (92)	342 (72)	276 (97)	241 (99)	1251 (70)
Ever attended school/madrasha	326 (79)	223 (52)	70 (14)	278 (97)	220 (83)	1117 (59)
Number in household (median (range))	6 (min: 2, max: 32)	7 (min: 2, max: 32)	7 (min: 2, max: 35)	4 (min: 2, max: 13)	5 (min: 2, max: 31)	
Electricity	179 (44)	420 (99)	451 (92)	283 (99)	218 (82)	1551 (83)
Piped water	10 (2)	272 (64)	137 (28)	244 (85)	31 (12)	694 (37)
Cell phone ownership	345 (84)	336 (79)	367 (75)	274 (96)	234 (88)	1556 (83)
Infant	**N=412**	**N=437**	**N=492**	**N=287**	**N=267**	**N=1895**
Male	187 (45)	204 (47)	229 (47)	141 (49)	133 (50)	894 (47)
Preterm	87 (21)	135 (31)	151 (31)	18 (6)	71 (27)	462 (24)
Low birthweight	108 (26)	86 (20)	189 (39)	46 (16)	49 (18)	478 (25)
Post birth massage	68 (17)	168 (39)	350 (71)	0 (0)	78 (30)	664 (35)
Post birth wash	53 (13)	410 (97)	485 (99)	171 (60)	204 (78)	1323 (71)
[φ]Proper cord care at birth	11 (3)	15 (4)	33 (8)	22 (8)	44 (18)	125 (7)
[δ]Age at breast milk supplementation (median days)	34 (min: 0, max: 59)	5 (min: 0, max: 64)	6 (min: 0, max: 61)	27 (min: 2, max: 61)	4 (min: 1, max: 48)	
Ever vaccinated (assessed at day 59 visit)	135/345 (39)	169/252 (67)	204/376 (54)	283/285 (99)	230/249 (93)	1021/1507 (68)
Received BCG	76 (56)	140 (83)	142 (70)	269 (95)	210 (91)	837 (82)
Received at least 1 oral polio vaccine	57 (42)	131 (78)	117 (57)	269 (95)	184 (80)	758 (74)
Received a diphtheria, tetanus, pertussis toxin containing vaccine	24 (18)	29 (17)	50 (25)	250 (88)	122 (53)	475 (47)
Received pneumococcal conjugate vaccine	0 (0)	4 (2)	0 (0)	0 (0)	3 (1)	7 (1)
Death rate among infants <60d (per 1000 live births)	0·05	0·11	0·11	0	0	0·07

***Received full antenatal package defined as: 1. receipt of at least 2 antenatal visits from a community health worker; AND 2. Receipt of 2 tetanus injections during current pregnancy, OR 1 tetanus injection and at least 4 shots pre-pregnancy, OR at least 5 tetanus injections pre-pregnancy; AND 3· Receipt of at least one iron tablet or dose or iron syrup during current pregnancy·**

[ζ]Poor nutritional status defined as mid upper arm circumference of less than 21·5 centimeters.

[δ]Age at breast milk supplementation defined as the age (days) at which any food or liquid other than breast milk was given to the baby.

[ʒ]Skilled birth attendant defined as a qualified doctor, nurse, midwife, or paramedic.

[φ]Proper cord care defined as use of an acceptable device for both cutting and tying the umbilical cord. Acceptable devices for cutting cords in the home must be boiled and be either thread, clip kit, knife, blade, or tongs. Acceptable devices for cutting cords in a hospital or health facility should be nurse/doctor's scissor or clip kit. Acceptable devices for tying cord in the home must be boiled and be either a clip, clip kit, thin rope brought by doctor, blade, or rubber band. Acceptable devices for tying a cord in a hospital or health facility is a clip or clip kit. In the home, antibiotic or antiseptic should be applied to the stump. In the hospital, antibiotic or antiseptic can be applied, or nothing can be applied.

Variables with >10% missing data: number of ANC visits (17%) and first birth (10%)

SM 5a. **List of the blood culture isolates determined as clinically non-significant by study criteria, Aetiology of Neonatal Infections in South Asia (ANISA) Study**

**Organism Name**	**Number of isolates**
Bacillus spp.	56
Brevundimonas spp.	1
Burkholderia spp.	1
Corynebacterium sp.	10
Diptheroid spp.	10
Kocuria spp.	2
Micrococcus spp.	19
Staphylococcus epidermidis	25
Staphylococcus saprophyticus	3
Staphylococcus spp.	73
Other Anaerobic bacteria	1
Others	5
**Total**	206

SM 5b. Characteristics of the blood culture isolates determined as non-significant through expert review in Aetiology of Neonatal Infections in South Asia (ANISA) Study

Study ID	Isolate 1	Age and sex (M-male, F-Female)	Clinical sign (s) presented at assessment	Drug susceptibility	Treatment received	Patient Outcome	Comment
1.	*S. infantarius.*	one day, M	hypothermia (92·0F), poor feeding and less movement	Sensitive to all common antibiotics	Unknown	Died	Extremely preterm infant with very low birth weight (1200g), clinical signs and symptoms presented from first day of life, consistent with the preterm and low birth weight babies; detected isolate naturally found in fermented food products and rarely known to cause neonatal sepsis.
2.	*Escherichia coli*	48 days, F	Fast breathing and severe chest indrawn	sensitive to common antibiotics	Inj. Ceftazidime and Amikacin	cured	Detection of multiple bacterial isolates from a case with only respiratory symptoms and without fever does not does not correlate.
3.	*M. catarrahlis*	20 days, F	Fast breathing	Sensitive to all common antibiotics	Oral amoxicillin	died	Insufficient and somewhat incompatible clinical signs, isolate was slow grower in culture media
4.	*P. luteola*	two days, M	Fever (100·8F), Fast breathing (72/m)	Sensitive to all common antibiotics	Inj. Penicillin Gentamicin and oral amoxicillin	cured	A baby who became symptom free within 48 hrs was very unlikely to have a *Pseudomonas* infection.
5.	*P. stutzeri*	44 days, M	Fever (101·5F), poor feeding	sensitive to common antibiotics	Inj. Penicillin Gentamicin	cured	A baby with *Pseudomonas* infection become symptom free within 48 hrs is very unlikely.
6.	*C. jejuni*	16 days, F	Fever (100·8F), poor feeding	sensitive to common antibiotics except ofloxacin	Inj. Penicillin and gentamicin	cured	Infection with *C. jejuni* isolate without diarrhea is unlikely, a conclusion further reinforced by the presence of mild clinical symptoms that disappeared within 48 hours.
7.	*C. jejuni*	two days, F	lethargic from first day	Sensitive to gentamicin but resistant to ampicillin	Inj. Penicillin and gentamicin and oral amoxicillin	cured	Sepsis by *C. jejuni* from day one of life is very unlikely, also diarrhea was not present.
8.	*Campylobacter sp.*	11 days, M	poor feeding	resistant to erythromycin	Inj. Penicillin and gentamicin	cured	LBW child (1900g), single clinical symptom, and no diarrhea is very unlikely to be *C. jejuni* infection.
9.	*C. jejuni*	38 days, M	severe chest indrawn	Resistant to ofloxacin	Inj. Penicillin and gentamicin	cured	Severe chest indrawing without fever and diarrhea is unlikely to be a infection with *C. jejuni*.
10.	*S. marcescens*	one day, M	poor feeding	Resistant to Ampicillin	Inj. Gentamicin and oral amoxicillin	cured	LBW (2350g) newborn with cephalhematoma and poor feeding on first day of life and improved from the next day is inconsistent with true *Serratia* infection.
11.	*C. jejuni*	one day, F	fever (100·8F) and Fast breathing	sensitive common antibiotics	Gentamicin and Ceftriaxone	cured	Sepsis by *C. jejuni* from day one of life is very unlikely, also diarrhea was not present. Possibly the baby had other infection.
12.	*B. cepacia*	39 days, M	poor feeding and fast breathing (80/m)	Resistant most common antibiotics including Amikacin, Ceftriaxone, and Netilmicin.	Unknown	cured	Multidrug resistant opportunistic pathogen, treated with inappropriate antibiotics and symptoms resolved within 24 hrs, is unlikely to be the causative pathogen.

13.	*E. coli*	48 days, F	fever (102·5F), fast breathing (81/m)	Resistant to Ampicillin Ceftriaxone Ceftazidime Cotrimoxazole Cefixime,	Oral Cefixime followed by Inj. Cefotaxime	cured	Multidrug resistant pathogen, treated with inappropriate antibiotics and symptoms resolved within three days, is unlikely to be the causative pathogen.
14.	*C. jejuni*	47 days, F	fever (100·8F) and fast breathing (72/m)	Resistant to Ofloxacin	Syp. Amoxicillin	cured	Organism grew after three days of antibiotic therapy and baby improved continuing with the same antibiotic. Also, baby did not have diarrhea, a common sign of C. jejuni infection
15.	*Campylobacter jejuni*	two days	Fever (100·7F Pseudomonas)	Resistant to Ofloxacin.	Inj. Ampicillin and oral azithromycin	cured	*C. jejuni* infection is very unlikely in such a young (2 days old) infant.
16.	*Pseudomonas sp.*	34 day	severe chest indrawn	Resistant to Ceftazidime, Aztreonam	oral cephalexin	cured	Clinical course and resistance pattern inconsistent with treatment features.
17.	*S. aureus*	13 day M	fast breathing and poor feeding	Resistant Cotrimoxazole, Penicillin, Fusidic.	Cefotaxime	cured	Organism grew after three days of antibiotic therapy and was sensitive to the antibiotics used.
18.	*Streptococcus mitis*	three days, F	Fever (101·8F)	Resistant to Ampicillin, Cefotaxime, Penicillin	Cefaclor	cured	Mild clinical course, improved with inappropriate antibiotic treatment.
19.	*Pseudomonas sp.*	one day, F	fever (100·6F) and fast breathing, poor feeding and observed convulsion	sensitive to common antibiotic	Azithromycin and unknown drug	cured	Clinical manifestations other than fever are consistent with perinatal asphyxia. Fever disappeared within 24 hours which is unlikely with Pseudomonas infection, Culture was also contaminated.
20.	*B. cepacia*	13 days, M	severe chest indrawn	Resistant to Ceftazidime, Tetracycline, Ofloxacin, Azithromycin	Inj. Ceftriaxone	cured	The patient received an antibiotic before blood collection and recovered with same antibiotic that the isolate was resistant to.
21.	*S. aureus*	27 days, M	severe chest indrawn	Resistant to penicillin	Amoxicillin drop		Mild clinical sign and rapid recovery despite using an inappropriate antibiotic.
22.	*S. epidermidis*	14 days, F	severe chest indrawn and fast breathing (66/m)	not available	Oral Cephalexin	cured	First blood culture was contaminated, second culture yielded Clostridium while receiving antibiotic and patient recovered with the same antibiotic.
23.	*C. jejuni*	six days, F	Hypothermia (93·3F)	Resistant to: Ampicillin, Ofloxacin.	Cephalexin	died	Presenting clinical signs were consistent with extreme prematurity (1000g) and low birth weight, also diarrhea was not present. Time to blood culture positivity was very long.
24.	*K. pneumoniae*	nine days, M	poor feeding	Resistant to common antibiotics including Amikacin, , Ceftriaxone, and Netilmicin,	Inj. Cefotaxime and Amikacin	cured	Rapid clinical recovery with antibiotics to which the isolates were resistant.
25.	*E. coli*	46 days, M	fever (103·0F)	Resistant to Ampicillin, Cefotaxime Ceftriaxone,	Inj. Amikacin and Cefotaxime	cured	The patient had single clinical sign and multiple bacterial isolate were identified from the blood. Detected organisms and clinical course and outcome are inconsistent. Although the isolates grow very fast in culture media, the blood culture positivity time were very long.

26.	*K. pneumoniae*	three days, M	lethargic and poor feeding	Resistant to all common antibiotics	Inj. Cefotaxime and Amikacin	cured	Patient recovered with an antibiotic to which the organism was resistant.
27.	*S. aureus*	56 days, M	lethargic and poor feeding	Resistant to Azithromycin, Ceftazidime, Ciprofloxacin, Penicillin	Inj. Cefotaxime	cured	Patient recovered within 3 days with an antibiotic to which the organism was resistant.
28.	*S. aureus*	one day, F	Fever (100·9), lethargic, poor feeding, convulsion	Resistant to: Vancomycin	Inj. Cefotaxime	cured	The illness pattern is not consistent with S. aureus infection, may have been infected with another pathogen susceptible to cefotaxime.
29.	*S. aureus*	11 days, M	lethargic, poor feeding, Fast breathing (662/m)	Resistant to: Ampicillin, Ceftazidime, Penicillin, and Vancomycin.	Inj. Piperacillin Tazobactam and Netilmicin	cured	Unusual susceptibility profile including resistance to vancomycin. The antibiotic being used for treatment has minimal/no activity against gram positive organisms specifically the MRSA.
30.	*Enterococcus sp.*	four days, F	poor feeding	Resistant to Ampicillin, Ceftriaxone, Penicillin	Inj. Cefotaxime	cured	Patient recovered, though the organism was resistant to the antibiotics being used for treatment.

SM 6. Detection of pathogens by TaqMan Array Cards (TAC) in specimens from possible serious bacterial infection episodes and healthy infants in each site, ANISA study, Aetiology of Neonatal Infections in South Asia (ANISA) Study

	Sylhet[2]						Karachi[3]						Matiari[4]					
	NP/OP[1] TAC			Blood TAC			NP/OP TAC			Blood TAC			NP/OP TAC			Blood TAC		
	Positive cases (%)	Positive healthy infants (%)	Odds Ratio	Positive cases (%)	Positive healthy infants (%)	Odds Ratio	Positive cases (%)	Positive healthy infants (%)	Odds Ratio	Positive cases (%)	Positive healthy infants (%)	Odds Ratio	Positive cases (%)	Positive healthy infants (%)	Odds Ratio	Positive cases (%)	Positive healthy infants (%)	Odds Ratio
**Number tested[5]**	**N = 1367**	**N = 412**		**N = 983**	**N = 344**		**N = 1235**	**N = 436**		**N = 1006**	**N = 370**		**N = 1344**	**N = 491**		**N = 1015**	**N = 458**	
**Pathogen**																		
Adenovirus	30(2·2)	12(2·9)	0·7				24(1·9)	5(1·1)	1·7				16(1·2)	11(2·2)	0·5			
Bordetella sp·	21(1·5)	6(1·5)	1·1				167(13·5)	46(10·6)	1·3				7(0·5)	2(0·4)	1·3			
Chlamydia pneumoniae	8(0·6)	2(0·5)	1·2				0(0)	0(0)	/				2(0·1)	1(0·2)	0·7			
Chlamydia trachomatis	8(0·6)	1(0·2)	2·4				0(0)	3(0·7)	0				4(0·3)	0(0)	/			
Cytomegalovirus	162(14·1)	44(12·3)	1·2				40(3·9)	29(6·8)	0·6				44(3·3)	22(4·8)	0·7			
Escherichia coli	501(36·6)	123(29·9)	1·4	24(2·4)	11(3·2)	0·8	229(18·5)	68(15·6)	1·2	8(0·8)	2(0·5)	1·5	223(16·6)	81(16·5)	1·0	5(0·5)	3(0·7)	0·8
Influenza A	21(1·5)	3(0·7)	2·1				10(0·8)	4(0·9)	0·9				7(0·5)	3(0·6)	0·9			
Influenza B	15(1·1)	0(0)	/				7(0·6)	2(0·5)	1·2				5(0·4)	1(0·2)	1·8			
Group A *streptococcus*				4(0·4)	0(0)	/				2(0·2)	0(0)	/				4(0·4)	0(0)	/
Group B *streptococcus*	267(19·5)	74(18·0)	1·1	7(0·7)	6(1·7)	0·4	51(4·1)	13(3·0)	1·4	0(0)	0(0)	/	32(2·4)	7(1·4)	1·7	5(0·5)	0(0)	/
pan-*Haemophilus influenzae*				17(1·7)	3(0·9)	2·0				7(0·7)	0(0)	/				6(0·6)	1(0·2)	2·7
Human metapneumovirus	11(0·8)	1(0·2)	3·3				7(0·6)	2(0·5)	1·2				1(0·1)	2(0·4)	0·2			
Human parechovirus	9(0·7)	5(1·2)	0·5				2(0·2)	4(0·9)	0·2				5(0·4)	2(0·4)	0·9			
Klebsiella pneumoniae	341(24·9)	101(24·5)	1·0	41(4·2)	22(6·4)	0·6	268(21·7)	127(29·1)	0·7	0(0)	0(0)	/	194(14·4)	51(10·4)	1·5	3(0·3)	0(0)	/
Mycoplasma pneumoniae	4(0·3)	0(0)	/				5(0·4)	1(0·2)	1·8				1(0·1)	0(0)	/			
Neisseria meningitidis				5(0·5)	2(0·6)	0·9				0(0)	0(0)	/				1(0·1)	1(0·2)	0·5
Parainfluenza virus type 1	9(0·7)	4(1·0)	0·7				5(0·4)	1(0·2)	1·8				5(0·4)	0(0)	/			
Parainfluenza virus type 2	3(0·2)	2(0·5)	0·5				0(0)	0(0)	/				3(0·2)	2(0·4)	0·5			
Parainfluenza virus type 3	31(2·3)	6(1·5)	1·6				15(1·2)	2(0·5)	2·7				5(0·4)	3(0·6)	0·6			
Pseudomonas aeruginosa				2(0·2)	3(0·9)	0·2				2(0·2)	0(0)	/				0(0)	1(0·2)	0
Respiratory syncytial virus	161(11·8)	3(0·7)	18·2				73(5·9)	4(0·9)	6·8				54(4·0)	7(1·4)	2·9			
Rhinovirus / Enterovirus	546(39·9)	190(46·1)	0·8	36(3·7)	6(1·7)	2·1	315(25·5)	142(32·6)	0·7	24(2·4)	10(2·7)	0·9	323(24·0)	158(32·2)	0·7	24(2·4)	6(1·3)	1·8
Rubella	4(0·3)	1(0·2)	1·2				2(0·2)	0(0)	/				9(0·7)	2(0·4)	1·6			
Salmonella spp·				19(1·9)	8(2·3)	0·8				15(1·5)	5(1·4)	1·1				30(3·0)	10(2·2)	1·4
Staphyloccocus aureus				7(0·7)	10(2·9)	0·2				2(0·2)	1(0·3)	0·7				5(0·5)	2(0·4)	1·1
Streptococcus pneumoniae	841(61·5)	248(60·2)	1·1	35(3·6)	10(2·9)	1·2	366(29·6)	126(28·9)	1·0	6(0·6)	2(0·5)	1·1	373(27·8)	160(32·6)	0·8	10(1·0)	3(0·7)	1·5
Ureaplasma spp·	199(14·6)	42(10·2)	1·5	5(0·5)	2(0·6)	0·9	99(8·0)	24(5·5)	1·5	6(0·6)	1(0·3)	2·2	96(7·1)	14(2·9)	2·6	6(0·6)	1(0·2)	2·7

[1]NP/OP=Nasopharyngeal/oropharyngeal
[2]Sylhet: NP/OP TAC: Number of cases tested for Cytomegalovirus N = 1152; Number of healthy infants tested for Cytomegalovirus N = 357; Blood TAC: Number of cases tested for *Neisseria meningitidis* N = 962
[3]Karachi: NP/OP TAC: Number of cases tested for Cytomegalovirus N = 1015; Number of healthy infants tested for Cytomegalovirus N = 429
[4]Matiari: NP/OP TAC: Number of cases tested for Cytomegalovirus N = 1321; Number of healthy infants tested for Cytomegalovirus N = 460
[5] All cases and healthy infants with at least one test result available are included in this table·

	Vellore						Odisha					
	NP/OP TAC			Blood TAC			NP/OP TAC			Blood TAC		
	**Positive cases (%)**	**Positive healthy infants (%)**	**Odds Ratio**	**Positive cases (%)**	**Positive healthy infants (%)**	**Odds Ratio**	**Positive cases (%)**	**Positive healthy infants (%)**	**Odds Ratio**	**Positive cases (%)**	**Positive healthy infants (%)**	**Odds Ratio**
**Number tested**	**N = 479**	**N = 287**		**N = 475**	**N = 281**		**N = 784**	**N = 267**		**N = 737**	**N = 264**	
**Pathogen**												
Adenovirus	1(0·2)	4(1·4)	0·1				4(0·5)	2(0·7)	0·7			
Bordetella spp.	18(3·8)	15(5·2)	0·7				21(2·7)	4(1·5)	1·8			
Chlamydia pneumoniae	0(0)	0(0)	/				1(0·1)	0(0)	/			
Chlamydia trachomatis	0(0)	0(0)	/				0(0)	1(0·4)	0			
Cytomegalovirus	26(5·4)	21(7·3)	0·7				91(11·6)	37(13·9)	0·8			
Escherichia coli	94(19·6)	70(24·4)	0·8	11(2·3)	9(3·2)	0·7	316(40·3)	119(44·6)	0·8	27(3·7)	7(2·7)	1·4
Influenza A	3(0·6)	1(0·3)	1·8				12(1·5)	2(0·7)	2·1			
Influenza B	0(0)	0(0)	/				4(0·5)	0(0)	/			
Group A *streptococcus*				2(0·4)	0(0)	/				2(0·3)	0(0)	/
Group B *streptococcus*	51(10·6)	18(6·3)	1·8	4(0·8)	0(0)	/	26(3·3)	11(4·1)	0·8	3(0·4)	0(0)	/
pan-*Haemophilus influenzae*				1(0·2)	1(0·4)	0·6				5(0·7)	1(0·4)	1·8
Human metapneumovirus	2(0·4)	0(0)	/				0(0)	1(0·4)	0			
Human parechovirus	1(0·2)	1(0·3)	0·6				3(0·4)	0(0)	/			
Klebsiella pneumoniae	122(25·5)	78(27·2)	0·9	13(2·7)	9(3·2)	0·9	224(28·6)	65(24·3)	1·2	20(2·7)	2(0·8)	3·7
Mycoplasma pneumoniae	0(0)	0(0)	/				0(0)	0(0)	/			
Neisseria meningitidis				0(0)	1(0·4)	0				1(0·1)	0(0)	/
Parainfluenza virus type 1	1(0·2)	2(0·7)	0·3				5(0·6)	0(0)	/			
Parainfluenza virus type 2	1(0·2)	2(0·7)	0·3				0(0)	1(0·4)	0			
Parainfluenza virus type 3	4(0·8)	2(0·7)	1·2				10(1·3)	3(1·1)	1·1			
Pseudomonas aeruginosa				3(0·6)	1(0·4)	1·8				12(1·6)	2(0·8)	2·2
Respiratory syncytial virus	36(7·5)	1(0·3)	23·2				78(9·9)	10(3·7)	2·8			
Rhinovirus/ Enterovirus	88(18·4)	65(22·6)	0·8	8(1·7)	4(1·4)	1·2	249(31·8)	89(33·3)	0·9	39(5·3)	23(8·7)	0·6
Rubella	1(0·2)	1(0·3)	0·6				4(0·5)	1(0·4)	1·4			
Salmonella spp.				5(1·1)	4(1·4)	0·7				9(1·2)	3(1·1)	1·1
Staphyloccocus aureus				10(2·1)	2(0·7)	3·0				5(0·7)	0(0)	/
Streptococcus pneumoniae	56(11·7)	33(11·5)	1·0	0(0)	3(1·1)	0	207(26·4)	61(22·8)	1·2	10(1·4)	2(0·8)	1·8
Ureaplasma spp.	44(9·2)	13(4·5)	2·1	0(0)	1(0·4)	0	128(16·3)	25(9·4)	1·9	2(0·3)	1(0·4)	0·7

SM 7. Detection of pathogens by TaqMan Array Cards (TAC) in blood and respiratory specimens from possible serious bacterial infection episodes and healthy infants stratified by age-at-onset[1] of infection, Aetiology of Neonatal Infections in South Asia (ANISA) Study

	Early-onset[2]						Late-onset[3]					
	NP/OP TAC			Blood TAC			NP/OP TAC			Blood TAC		
	Positive cases (%)	Positive healthy infants (%)	Odds Ratio	Positive cases (%)	Positive healthy infants (%)	Odds Ratio	Positive cases (%)	Positive healthy infants (%)	Odds Ratio	Positive cases (%)	Positive healthy infants (%)	Odds Ratio
**Number Tested[4]**	**N = 2081**	**N = 561**		**N = 1645**	**N = 520**		**N = 3128**	**N = 1332**		**N = 2571**	**N = 1197**	
**Pathogen**												
Adenovirus	19(0·9)	5(0·9)	1·0				56(1·8)	29(2·2)	0·8			
Bordetella spp.	104(5·0)	18(3·2)	1·6				130(4·2)	55(4·1)	1·0			
Chlamydia pneumoniae	1(0·05)	1(0·2)	0·3				10(0·3)	2(0·2)	2·1			
Chlamydia trachomatis	0(0)	0(0)	/				12(0·4)	5(0·4)	1·0			
Cytomegalovirus	60(3·2)	14(2·6)	1·2				303(10·6)	139(10·9)	1·0			
Escherichia coli	429(20·6)	120(21·4)	1·0	23(1·4)	12(2·3)	0·6	934(29·9)	341(25·6)	1·2	52(2)	20(1·7)	1·2
Influenza A	3(0·1)	2(0·4)	0·4				50(1·6)	11(0·8)	2·0			
Influenza B	3(0·1)	1(0·2)	0·8				28(0·9)	2(0·2)	6·0			
Group A *streptococcus*				3(0·2)	0(0)	/				11(0·4)	0(0)	/
Group B *streptococcus*	183(8·8)	40(7·1)	1·3	11(0·7)	3(0·6)	1·2	244(7·8)	83(6·2)	1·3	8(0·3)	3(0·3)	1·2
pan-*Haemophilus influenzae*				4(0·2)	2(0·4)	0·6				32(1·2)	4(0·3)	3·8
Human metapneumovirus	2(0·1)	0(0)	/				19(0·6)	6(0·5)	1·4			
Human parechovirus	3(0·1)	3(0·5)	0·3				17(0·5)	9(0·7)	0·8			
Klebsiella pneumoniae	356(17·1)	93(16·6)	1·0	28(1·7)	7(1·3)	1·3	793(25·4)	329(24·7)	1·0	49(1·9)	26(2·2)	0·9
Mycoplasma pneumoniae	1(0)	0(0)	/				9(0·3)	1(0·1)	3·8			
Neisseria meningitides				0(0)	1(0·2)	0				7(0·3)	3(0·3)	1·1
Parainfluenza virus type 1	1(0)	0(0)	/				24(0·8)	7(0·5)	1·5			
Parainfluenza virus type 2	0(0)	0(0)	/				7(0·2)	7(0·5)	0·4			
Parainfluenza virus type 3	0(0)	1(0·2)	0				65(2·1)	15(1·1)	1·9			
Pseudomonas aeruginosa				7(0·4)	2(0·4)	1·1				12(0·5)	5(0·4)	1·1
Respiratory syncytial virus	10(0·5)	4(0·7)	0·7				392(12·5)	21(1·6)	8·9			
Rhinovirus / Enterovirus	116(5·6)	56(10·0)	0·5	2(0·1)	1(0·2)	0·6	1405(44·9)	588(44·1)	1·0	129(5)	48(4)	1·3
Rubella	6(0·3)	1(0·2)	1·6				14(0·4)	4(0·3)	1·5			
Salmonella spp.				32(1·9)	9(1·7)	1·1				46(1·8)	21(1·8)	1·0
Staphyloccocus aureus				10(0·6)	5(1·0)	0·6				19(0·7)	10(0·8)	0·9
Streptococcus pneumoniae	277(13·3)	62(11·1)	1·2	10(0·6)	4(0·8)	0·8	1566(50·1)	566(42·5)	1·4	51(2)	16(1·3)	1·5
Ureaplasma spp.	239(11·5)	24(4·3)	2·9	7(0·4)	1(0·2)	2·2	327(10·5)	94(7·1)	1·5	12(0·5)	5(0·4)	1·1

[1]Early-onset: Onset on day 0-2 of life; Late-onset: Onset on day 3 of life or later

[2]Early-onset: NP/OP TAC: Number of cases tested for Cytomegalovirus N = 1896; Number of healthy infants tested for Cytomegalovirus N = 530; Blood TAC: Number of cases tested for Neisseria meningitidis N = 1638

[3]Late-onset: NP/OP TAC: Number of cases tested for Cytomegalovirus N = 2855; Number of healthy infants tested for Cytomegalovirus N = 1270; Blood TAC: Number of cases tested for *Neisseria meningitidis* N = 2557

[4] All cases and healthy infants with at least one test result available are included in this table

SM 8. Detection of pathogens by TaqMan Array Cards (TAC) in blood and respiratory specimens from infants that died, Aetiology of Neonatal Infections in South Asia (ANISA) Study

	NP/OP TAC [1,2]			Blood TAC[3]		
	Positive cases (%)	Positive healthy infants (%)	Odds Ratio	Positive cases (%)	Positive healthy infants (%)	Odds Ratio
**Number tested[4]**	**N = 333**	**N = 1893**		**N = 201**	**N = 1717**	
**Pathogen**						
Adenovirus	5(1·5)	34(1·8)	0·8			
Bordetella spp.	16(4·8)	73(3·9)	1·3			
Chlamydia pneumoniae	2(0·6)	3(0·2)	3·8			
Chlamydia trachomatis	0(0)	5(0·3)	0			
Cytomegalovirus	28(9·0)	153(8·5)	1·1			
Escherichia coli	127(38·1)	461(24·4)	1·9	8(4·0)	32(1·9)	2·2
Influenza A	0(0)	13(0·7)	0			
Influenza B	1(0·3)	3(0·2)	1·9			
Group A *streptococcus*				1(0·5)	0(0)	/
Group B *streptococcus*	47(14·1)	123(6·5)	2·4	1(0·5)	6(0·3)	1·4
pan-*Haemophilus influenzae*				6(3·0)	6(0·3)	8·8
Human metapneumovirus	0(0)	6(0·3)	0			
Human parechovirus	1(0·3)	12(0·6)	0·5			
Klebsiella pneumoniae	109(32·7)	422(22·3)	1·7	7(3·5)	33(1·9)	1·8
Mycoplasma pneumoniae	2(0·6)	1(0·1)	11·4			
Neisseria meningitidis				2(1·0)	4(0·2)	4·3
Parainfluenza virus type 1	0(0)	7(0·4)	0			
Parainfluenza virus type 2	0(0)	7(0·4)	0			
Parainfluenza virus type 3	0(0)	16(0·8)	0			
Pseudomonas aeruginosa				0(0)	7(0·4)	0
Respiratory syncytial virus	18(5·4)	25(1·3)	4·3			
Rhinovirus / Enterovirus	56(16·8)	644(34·0)	0·4	4(2·0)	49(2·9)	0·7
Rubella virus	4(1·2)	5(0·3)	4·6			
Salmonella spp.				5(2·5)	30(1·7)	1·4
Staphyloccocus aureus				1(0·5)	15(0·9)	0·6
Streptococcus pneumoniae	110(33·0)	628(33·2)	1·0	7(3·5)	20(1·2)	3·1
Ureaplasma spp.	66(19·8)	118(6·2)	3·7	1(0·5)	6(0·3)	1·4

[1]NP/OP=Nasopharyngeal/oropharyngeal
[2]NP/OP TAC: Number of cases tested for Cytomegalovirus N = 311; Number of healthy infants tested for Cytomegalovirus N = 1800
[3]Blood TAC: Number of cases tested for *Neisseria meningitidis* N = 199
[4] All cases and healthy infants with at least one test result available are included in this table

By Site

	Sylhet[2]						Karachi[3]						Matiari[4]					
	NP/OP TAC [1]			Blood TAC			NP/OP TAC			Blood TAC			NP/OP TAC			Blood TAC		
	Positive cases (%)	Positive healthy infants (%)	Odds Ratio	Positive cases (%)	Positive healthy infants (%)	Odds Ratio	Positive cases (%)	Positive healthy infants (%)	Odds Ratio	Positive cases (%)	Positive healthy infants (%)	Odds Ratio	Positive cases (%)	Positive healthy infants (%)	Odds Ratio	Positive cases (%)	Positive healthy infants (%)	Odds Ratio
**Number tested[5]**	**N = 129**	**N = 412**		**N = 70**	**N = 344**		**N = 72**	**N = 436**		**N = 43**	**N = 370**		**N = 88**	**N = 491**		**N = 50**	**N = 458**	
**Pathogen**																		
Adenovirus	2(1·6)	12(2·9)	0·5				2(2·8)	5(1·1)	2·5				1(1·1)	11(2·2)	0·5			
Bordetella spp.	2(1·6)	6(1·5)	1·1				13(18·1)	46(10·6)	1·9				1(1·1)	2(0·4)	2·8			
Chlamydia pneumoniae	1(0·8)	2(0·5)	1·6				0(0)	0(0)	/				1(1·1)	1(0·2)	5·6			
Chlamydia trachomatis	0(0)	1(0·2)	0				0(0)	3(0·7)	0				0(0)	0(0)	/			
Cytomegalovirus	16(13·4)	44(12·3)	1·1				2(3·2)	29(6·8)	0·5				4(4·7)	22(4·8)	1·0			
Escherichia coli	62(48·1)	123(29·9)	2·2	5(7·1)	11(3·2)	2·3	25(34·7)	68(15·6)	2·9	0(0)	2(0·5)	0	20(22·7)	81(16·5)	1·5	0(0)	3(0·7)	0
Influenza A	0(0)	3(0·7)	0				0(0)	4(0·9)	0				0(0)	3(0·6)	0			
Influenza B	1(0·8)	0(0)	/				0(0)	2(0·5)	0				0(0)	1(0·2)	0			
Group A *streptococcus*				0(0)	0(0)	/				0(0)	0(0)	/				1(2)	0(0)	/
Group B *streptococcus*	36(27·9)	74(18·0	1·8	1(1·4)	6(1·7)	0·8	7(9·7)	13(3·0)	3·5	0(0)	0(0)	/	2(2·3)	7(1·4)	1·6	0(0)	0(0)	/
pan-*Haemophilus influenzae*				4(5·7)	3(0·9)	6·9				1(2·3)	0(0)	/				1(2)	1(0·2)	9·3
Human metapneumovirus	0(0)	1(0·2)	0				0(0)	2(0·5)	0				0(0)	2(0·4)	0			
Human parechovirus	0(0)	5(1·2)	0				0(0)	4(0·9)	0				0(0)	2(0·4)	0			
Klebsiella pneumoniae	40(31·0)	101(24·5)	1·4	3(4·3)	22(6·4)	0·7	26(36·1)	127(29·1)	1·4	0(0)	0(0)	/	16(18·2)	51(10·4)	1·9	0(0)	0(0)	/
Mycoplasma pneumoniae	1(0·8)	0(0)	/				0(0)	1(0·2)	0				1(1·1)	0(0)	/			
Neisseria meningitidis				1(1·5)	2(0·6)	2·6				0(0)	0(0)	/				1(2)	1(0·2)	9·3
Parainfluenza virus type 1	0(0)	4(1)	0				0(0)	1(0·2)	0				0(0)	0(0)	/			
Parainfluenza virus type 2	0(0)	2(0·5)	0				0(0)	0(0)	/				0(0)	2(0·4)	0			
Parainfluenza virus type 3	0(0)	6(1·5)	0				0(0)	2(0·5)	0				0(0)	3(0·6)	0			
Pseudomonas aeruginosa				0(0)	3(0·9)	0				0(0)	0(0)	/				0(0)	1(0·2)	0
Respiratory syncytial virus	10(7·8)	3(0·7)	11·5				3(4·2)	4(0·9)	4·7				5(5·7)	7(1·4)	4·2			
Rhinovirus / Enterovirus	32(24·8)	190(46·1)	0·4	0(0)	6(1·7)	0	7(9·7)	142(32·6)	0·2	0(0)	10(2·7)	0	11(12·5)	158(32·2)	0·3	2(4)	6(1·3)	3·1
Rubella	0(0)	1(0·2)	0				1(1·4)	0(0)	/				2(2·3)	2(0·4)	5·7			
Salmonella spp.				1(1·4)	8(2·3)	0·6				1(2·3)	5(1·4)	1·7				2(4)	10(2·2)	1·9
Staphyloccocus aureus				1(1·4)	10(2·9)	0·5				0(0)	1(0·3)	0				0(0)	2(0·4)	0
Streptococcus pneumoniae	61(47·3)	248(60·2)	0·6	4(5·7)	10(2·9)	2·0	18(25·0)	126(28·9)	0·8	0(0)	2(0·5)	0	25(28·4)	160(32·6)	0·8	3(6)	3(0·7)	9·7
Ureaplasma spp.	30(23·3)	42(10·2)	2·7	0(0)	2(0·6)	0	7(9·7)	24(5·5)	1·8	0(0)	1(0·3)	0	12(13·6)	14(2·9)	5·4	1(2)	1(0·2)	9·3

[1]NP/OP=Nasopharyngeal/oropharyngeal
[2]Sylhet: NP/OP TAC: Number of cases tested for Cytomegalovirus N = 119; Number of healthy infants tested for Cytomegalovirus N = 357; Blood TAC: Number of cases tested for *Neisseria meningitidis* N = 68
[3]Karachi: NP/OP TAC: Number of cases tested for Cytomegalovirus N = 62; Number of healthy infants tested for Cytomegalovirus N = 429

[4]Matiari: NP/OP TAC: Number of cases tested for Cytomegalovirus N = 86; Number of healthy infants tested for Cytomegalovirus N = 460
[5] All cases and healthy infants with at least one test result available are included in this table·

	Vellore						Odisha					
	NP/OP TAC			Blood TAC			NP/OP TAC			Blood TAC		
	**Positive cases (%)**	**Positive healthy infants (%)**	**Odds Ratio**	**Positive cases (%)**	**Positive healthy infants (%)**	**Odds Ratio**	**Positive cases (%)**	**Positive healthy infants (%)**	**Odds Ratio**	**Positive cases (%)**	**Positive healthy infants (%)**	**Odds Ratio**
**Number tested**	**N = 8**	**N = 287**		**N = 7**	**N = 281**		**N = 36**	**N = 267**		**N = 31**	**N = 264**	
**Pathogen**												
Adenovirus	0(0)	4(1·4)	0				0(0)	2(0·7)	0			
Bordetella spp.	0(0)	15(5·2)	0				0(0)	4(1·5)	0			
Chlamydia pneumoniae	0(0)	0(0)	/				0(0)	0(0)	/			
Chlamydia trachomatis	0(0)	0(0)	/				0(0)	1(0·4)	0			
Cytomegalovirus	0(0)	21(7·3)	0				6(16·7)	37(13·9)	1·2			
Escherichia coli	3(37·5)	70(24·4)	1·9	1(14·3)	9(3·2)	5·0	17(47·2)	119(44·6)	1·1	2(6·5)	7(2·7)	2·5
Influenza A	0(0)	1(0·3)	0				0(0)	2(0·7)	0			
Influenza B	0(0)	0(0)	/				0(0)	0(0)	/			
Group A streptococcus				0(0)	0(0)	/				0(0)	0(0)	/
Group B streptococcus	1(12·5)	18(6·3)	2·1	0(0)	0(0)	/	1(2·8)	11(4·1)	0·7	0(0)	0(0)	/
pan-*Haemophilus influenzae*				0(0)	1(0·4)	0				0(0)	1(0·4)	0
Human metapneumovirus	0(0)	0(0)	/				0(0)	1(0·4)	0			
Human parechovirus	1(12·5)	1(0·3)	40·9				0(0)	0(0)	/			
Klebsiella pneumoniae	4(50·0)	78(27·2)	2·7	3(42·9)	9(3·2)	22·7	23(63·9)	65(24·3)	5·5	1(3·2)	2(0·8)	4·4
Mycoplasma pneumoniae	0(0)	0(0)	/				0(0)	0(0)				
Neisseria meningitidis				0(0)	1(0·4)	0				0(0)	0(0)	/
Parainfluenza virus type 1	0(0)	2(0·7)	0				0(0)	0(0)				
Parainfluenza virus type 2	0(0)	2(0·7)	0				0(0)	1(0·4)	0			
Parainfluenza virus type 3	0(0)	2(0·7)	0				0(0)	3(1·1)	0			
Pseudomonas aeruginosa				0(0)	1(0·4)	0				0(0)	2(0·8)	0
Respiratory syncytial virus	0(0)	1(0·3)	0				0(0)	10(3·7)	0			
Rhinovirus / Enterovirus	1(12·5)	65(22·6)	0·5	1(14·3)	4(1·4)	11·5	5(13·9)	89(33·3)	0·3	1(3·2)	23(8·7)	0·3
Rubella	0(0)	1(0·3)	0				1(2·8)	1(0·4)	7·6			
Salmonella spp.				0(0)	4(1·4)	0				1(3·2)	3(1·1)	2·9
Staphyloccocus aureus				0(0)	2(0·7)	0				0(0)	0(0)	/
Streptococcus pneumoniae	2(25·0)	33(11·5)	2·6	0(0)	3(1·1)	0	4(11·1)	61(22·8)	0·4	0(0)	2(0·8)	0
Ureaplasma spp.	2(25·0)	13(4·5)	7·0	0(0)	1(0·4)	0	15(41·7)	25(9·4)	6·9	0(0)	1(0·4)	0

SM 9. Estimates of pathogen-specific true positive rates for blood culture and TaqMan Array Cards (TAC) of blood and respiratory samples from a partial latent class attribution model used in Aetiology of Neonatal Infections in South Asia (ANISA) Study

	Blood TAC	NP/OP TAC	Blood Culture
Pathogen	Mean (% (2·5Q, 97·5Q))	Mean (% (2·5Q, 97·5Q))	Mean (% (2·5Q, 97·5Q))
Adenovirus		82·52(48·39,99·95)	
Bordetella spp.		85·49(49·86,99·98)	
Chlamydia pneumoniae		81·46(44·26,99·97)	
Chlamydia trachomatis		74·14(41·85,99·92)	
Cytomegalovirus		89·95(72·16,99·98)	
Escherichia coli	38·73(24·94,56·83)	73·30(67·77,80·41)	28·35(14·79,48·49)
Influenza A		81·48(44·17,99·97)	
Influenza B		81·06(45·57,99·97)	
Group A *Streptococcus*	78·05(51·52,95·38)		83·18(55·96,98·68)
Group B *Streptococcus*	29·8(20·3,52·16)	72·88(52·68,91·41)	12·81(4·23,27·25)
pan-Haemophilus influenzae	75·57(32·34,99·93)		
Human metapneumovirus		73·99(42·39,99·9)	
Human parechovirus		77·47(42·17,99·94)	
Klebsiella pneumoniae	31·20(23·03,46·57)	73·16(64·04,83·19)	21·72(11·71,37·51)
Mycoplasma pneumoniae		78·18(42·24,99·96)	
Neisseria meningitides	62·51(25·96,95·84)		57·3(21·48,92·39)
Parainfluenza virus type 1		78·65(42·71,99·93)	
Parainfluenza virus type 2		75·88(41·77,99·94)	
Parainfluenza virus type 3		85·18(50·67,99·98)	
Pseudomonas aeruginosa	47·04(21·19,89·32)		14·18(0·96,45·2)
Respiratory syncytial virus		95·86(82,100)	
Rhinovirus / Enterovirus	76·40(44·87,99·78)	78·64(75·11,86·33)	
Rubella		79·07(43·3,99·96)	
Salmonella spp.	42·53(23·25,74·18)		4·36(0·57,13·66)
Staphyloccocus aureus	34·53(21·54,54·88)		28·65(12·56,52·13)
Streptococcus pneumoniae	63·54(33·72,94·08)	83·62(75·6,93·95)	10·15(3·05,22·21)
Ureaplasma spp.	22·19(20·05,27·63)	61·54(48·34,76·24)	

[1] See SM1 for details regarding the ANISA partial latent class model methodology
[2] True positive rate is defined as the proportion of positive test results for a pathogen-specific test among episodes attributed to that pathogen

SM 10. Estimates of pathogen-specific false positive rates for TaqMan Array Cards (TAC) of blood and respiratory samples by study site from a partial latent class attribution model used in ANISA study

**Blood TAC**	**Sylhet**	**Karachi**	**Matiari**	**Vellore**	**Odisha**
Pathogen	Mean(% (2·5Q, 97·5Q))	Mean(% (2·5Q, 97·5Q))	Mean(% (2·5Q, 97·5Q))	Mean(% (2·5Q, 97·5Q))	Mean(% (2·5Q, 97·5Q))
Escherichia coli	2·41(1·98,2·88)	0·31(0·13,0·62)	0·34(0·22,0·53)	2·14(1·72,2·53)	2·22(1·54,2·7)
Group A *Streptococcus*	0·01(0,0·02)	0(0,0·01)	0(0,0·01)	0(0,0·02)	
Group B *Streptococcus*	0·99(0·81,1·17)	0·08(0·06,0·1)	0·06(0,0·21)	0(0,0·02)	0·01(0,0·02)
pan-*Haemophilus influenzae*		0·01(0,0·11)	0·07(0·04,0·17)		0·19(0·08,0·51)
Klebsiella pneumoniae	4·66(4·25,5·05)	0·17(0·15,0·2)	0·13(0·09,0·2)	2·09(1·67,2·48)	0·82(0·52,1·19)
Neisseria meningitides	0·31(0·27,0·47)	0(0,0·01)			
Pseudomonas aeruginosa			0·1(0·08,0·11)	0·19(0·11,0·28)	1·09(0·75,1·32)
Rhinovirus / Enterovirus	1·07(0·62,1·6)	2·68(2·15,3·05)	1·21(0·87,1·65)	1·16(0·76,1·65)	6·12(5·03,7·04)
Salmonella spp.	1·5(1·02,2·13)	0·69(0·38,1·07)	2·28(1·69,2·59)	1·06(0·71,1·38)	1·13(0·75,1·49)
Staphyloccocus aureus	1·48(1·22,1·71)		0·44(0·34,0·55)	0·53(0·18,0·97)	0·04(0,0·24)
Streptococcus pneumoniae	1·45(1·1,1·92)	0·28(0·15,0·4)	0·31(0·22,0·44)	0·36(0·26,0·49)	0·85(0·28,1·29)
Ureaplasma spp.	0·37(0·18,0·56)	0·09(0·05,0·26)	0·21(0·15,0·39)	0·11(0·07,0·15)	0·11(0·08,0·2)

**NP/OP TAC**	**Sylhet**	**Karachi**	**Matiari**	**Vellore**	**Odisha**
Pathogen	Mean(% (2·5Q, 97·5Q))	Mean(% (2·5Q, 97·5Q))	Mean(% (2·5Q, 97·5Q))	Mean(% (2·5Q, 97·5Q))	Mean(% (2·5Q, 97·5Q))
Adenovirus	1·89(1·35,2·51)	0·88(0·46,1·3)	1·15(0·9,1·43)		
Bordetella spp.	1·62(1·03,2·13)	12·64(11·37,13·55)	0·48(0·39,0·53)	3·8(3·24,4·32)	1·34(0·98,1·7)
Chlamydia pneumoniae	0·16(0·09,0·3)				
Chlamydia trachomatis	0·07(0·04,0·21)		0·03(0,0·15)		
Cytomegalovirus	13·24(10·66,14·97)	4·62(4·18,5·03)	3·31(2·6,3·83)	4·08(3·35,5·04)	11·1(9·45,12·3)
Escherichia coli	35·14(33·96,36·29)	16·63(15·87,17·29)	16·54(15·93,17·12)	20·17(18·96,21·25)	38·81(37·46,40·09)
Influenza A	0·65(0·28,1·11)	0·49(0·28,0·83)	0·49(0·25,0·67)		0·96(0·39,1·36)
Influenza B	0·02(0,0·14)	0·49(0·28,0·64)	0·09(0·04,0·22)		0·01(0,0·02)
Group B *Streptococcus*	18·62(17·57,19·63)	3·69(3·34,3·99)	1·76(1·41,2·11)	8·25(6·83,9·35)	4·01(3·5,4·49)
Human metapneumovirus	0·07(0·05,0·18)	0·24(0·12,0·38)		0·04(0,0·23)	
Human parechovirus	0·62(0·41,0·83)				0·12(0·09,0·15)
Klebsiella pneumoniae	24·97(23·89,25·98)	23·81(23·18,24·42)	13·04(12·43,13·57)	25·46(24·24,26·56)	23·64(22·07,25·04)
Mycoplasma pneumoniae					
Parainfluenza virus type 1	0·27(0·17,0·62)	0·17(0·09,0·34)	0·05(0,0·2)		0·02(0,0·23)
Parainfluenza virus type 2	0·2(0·1,0·36)		0·14(0·08,0·21)		
Parainfluenza virus type 3	0·99(0·66,1·33)	0·47(0·19,0·83)	0·21(0·13,0·37)	0·51(0·28,0·87)	1·26(0·73,1·58)
Respiratory syncytial virus	0·87(0·37,1·76)	0·62(0·35,1)	1·11(0·68,1·71)	0·42(0·15,1·02)	3·81(2·32,5·94)
Rhinovirus / Enterovirus	41·3(40·16,42·39)	25·35(24·72,25·99)	25·22(24·6,25·81)	17·97(16·99,18·89)	34·46(33·08,35·65)
Rubella			0·13(0·08,0·24)		
Streptococcus pneumoniae	56·6(53·98,59·19)	26·23(25·57,26·87)	27·41(26·76,28·06)	9·58(8·72,10·38)	26·14(24·85,27·43)
Ureaplasma spp.	12·08(10·88,13·1)	6·25(5·35,6·96)	3·07(2·01,4·21)	6·65(5·77,7·36)	12·61(11·2,13·77)

[1] See SM1 for details regarding the ANISA partial latent class model methodology
[2] False positive rate is defined as the proportion of positive results for a pathogen-specific test among episodes that were not attributed to that pathogen
[3] False positive rates reported represent the average value across covariates (age, time of enrollment, outcome) for a given site

SM 11: Sensitivity analysis of the influence of specimen capture from young infants who died on the overall pathogen prevalence distribution estimated by the partial latent class attribution model among infants with possible serious bacterial infection, Aetiology of Neonatal Infections in South Asia (ANISA) Study

Pathogen	Unadjusted proportion[1] (%)	Adjusted pathogen proportion (%) given varying assumptions about the percentage of deaths fulfilling the pSBI case definition[2,3]			
		100%	70%	50%	30%
Adenovirus	0·50(0·26, 0·92)	0·31(0·16, 0·57)	0·35(0·19, 0·65)	0·39(0·21, 0·72)	0·44(0·23, 0·81)
Bordetella spp.	0·80(0·41, 1·58)	0·99(0·44, 2·17)	0·95(0·45, 1·99)	0·91(0·44, 1·86)	0·87(0·44, 1·71)
Chlamydia pneumoniae	0·09(0·04, 0·18)	0·06(0·03, 0·11)	0·06(0·03, 0·13)	0·07(0·03, 0·14)	0·08(0·04, 0·16)
Chlamydia trachomatis	0·25(0·14, 0·49)	0·15(0·08, 0·29)	0·18(0·10, 0·34)	0·20(0·11, 0·38)	0·22(0·12, 0·43)
Cytomegalovirus	0·83(0·36, 1·53)	0·65(0·31, 1·14)	0·69(0·32, 1·23)	0·73(0·34, 1·31)	0·78(0·35, 1·40)
Escherichia coli	1·71(1·05, 2·62)	3·90(2·45, 5·80)	3·32(2·09, 4·91)	2·85(1·78, 4·23)	2·28(1·41, 3·45)
Influenza A	0·51(0·24, 0·94)	0·33(0·15, 0·61)	0·38(0·17, 0·69)	0·41(0·19, 0·76)	0·46(0·21, 0·85)
Influenza B	0·53(0·38, 0·92)	0·36(0·26, 0·62)	0·40(0·29, 0·69)	0·44(0·31, 0·76)	0·48(0·34, 0·83)
Group A *Streptococcus*	0·30(0·27, 0·35)	0·19(0·18, 0·23)	0·22(0·20, 0·26)	0·24(0·22, 0·28)	0·27(0·24, 0·31)
Group B *Streptococcus*	1·12(0·65, 1·71)	1·64(0·92, 2·61)	1·51(0·86, 2·37)	1·40(0·80, 2·17)	1·26(0·73, 1·94)
pan-*Haemophilus influenzae*	0·44(0·25, 0·93)	0·30(0·16, 0·62)	0·33(0·18, 0·70)	0·36(0·20, 0·76)	0·40(0·22, 0·84)
Human metapneumovirus	0·41(0·27, 0·73)	0·28(0·17, 0·50)	0·31(0·20, 0·55)	0·34(0·21, 0·60)	0·37(0·24, 0·66)
Human parechovirus	0·17(0·09, 0·33)	0·13(0·07, 0·25)	0·14(0·08, 0·28)	0·15(0·08, 0·29)	0·16(0·08, 0·31)
Klebsiella pneumoniae	1·79(1·17, 2·49)	3·17(2·04, 4·45)	2·75(1·79, 3·86)	2·43(1·60, 3·40)	2·06(1·36, 2·87)
Mycoplasma pneumoniae	0(0, 0)	0(0, 0)	0(0, 0)	0(0, 0)	0(0, 0)
Neisseria meningitidis	0·19(0·12, 0·31)	0·11(0·08, 0·19)	0·13(0·09, 0·22)	0·15(0·10, 0·25)	0·16(0·11, 0·28)
Parainfluenza virus type 1	0·49(0·31, 0·87)	0·34(0·22, 0·62)	0·38(0·24, 0·68)	0·41(0·26, 0·73)	0·45(0·29, 0·80)
Parainfluenza virus type 2	0·07(0·03, 0·15)	0·04(0·02, 0·09)	0·05(0·02, 0·11)	0·05(0·03, 0·12)	0·06(0·03, 0·13)
Parainfluenza virus type 3	0·70(0·45, 1·21)	0·46(0·30, 0·80)	0·52(0·33, 0·90)	0·57(0·37, 0·99)	0·63(0·41, 1·09)
Pseudomonas aeruginosa	0·28(0·13, 0·62)	0·58(0·28, 1·35)	0·51(0·24, 1·17)	0·46(0·21, 1·03)	0·38(0·18, 0·83)
Respiratory syncytial virus	6·48(5·81, 7·59)	5·31(4·69, 6·29)	5·61(5·00, 6·64)	5·87(5·24, 6·93)	6·18(5·53, 7·24)
Rhinovirus / Enterovirus	1·36(0·83, 2·37)	1·42(0·93, 2·23)	1·40(0·92, 2·26)	1·39(0·89, 2·29)	1·38(0·87, 2·32)
Rubella	0·14(0·08, 0·26)	0·08(0·05, 0·15)	0·09(0·05, 0·18)	0·10(0·06, 0·20)	0·12(0·07, 0·23)
Salmonella spp.	1·28(0·53, 2·52)	0·84(0·35, 1·64)	0·94(0·39, 1·86)	1·03(0·43, 2·04)	1·15(0·47, 2·26)
Staphyloccocus aureus	1·05(0·63, 1·68)	0·90(0·53, 1·42)	0·94(0·56, 1·50)	0·97(0·58, 1·56)	1·01(0·61, 1·63)
Streptococcus pneumoniae	1·15(0·70, 1·98)	1·86(1·11, 3·15)	1·69(1·02, 2·87)	1·55(0·94, 2·60)	1·37(0·83, 2·31)
Ureaplasma spp.	2·82(1·93, 3·77)	4·28(2·73, 5·91)	3·92(2·58, 5·31)	3·62(2·44, 4·87)	3·25(2·23, 4·29)
Other Blood Culture[4]	2·57(2·05, 3·11)	5·10(3·99, 6·32)	4·48(3·53, 5·52)	3·97(3·16, 4·86)	3·32(2·66, 4·04)
Other/None[5]	71·99(68·72, 74·91)	66·23(61·65, 70·33)	67·72(63·62, 71·41)	68·93(65·14, 72·31)	70·4(66·96, 73·53)

1 See SM1 for details regarding the ANISA partial latent class model methodology
2 Of 3061 deaths, 1684 registered and 1377 unregistered, specimens were available for 349 deaths. It's possible that up to 100% of these deaths met the pSBI case definition. Assuming that the distribution of pathogen proportions was the same among deaths with specimens as without specimens, we estimated the overall pathogen proportion under four different scenarios (30%, 50%, 70%, and 100% of deaths meeting the pSBI case definition)
3 For adjusted proportion estimates, all deaths meeting the pSBI case definition were assumed to have specimens with laboratory results available
4 The pathogen class 'Other Blood Culture' includes all bacteria that grew on blood culture but did not have an associated assay on the ANISA molecular diagnostic panel
5 The pathogen class 'Other/None' includes any pSBI episode that was not attributed by the partial latent class model (see text for details) to one of the evaluated ANISA pathogen classes

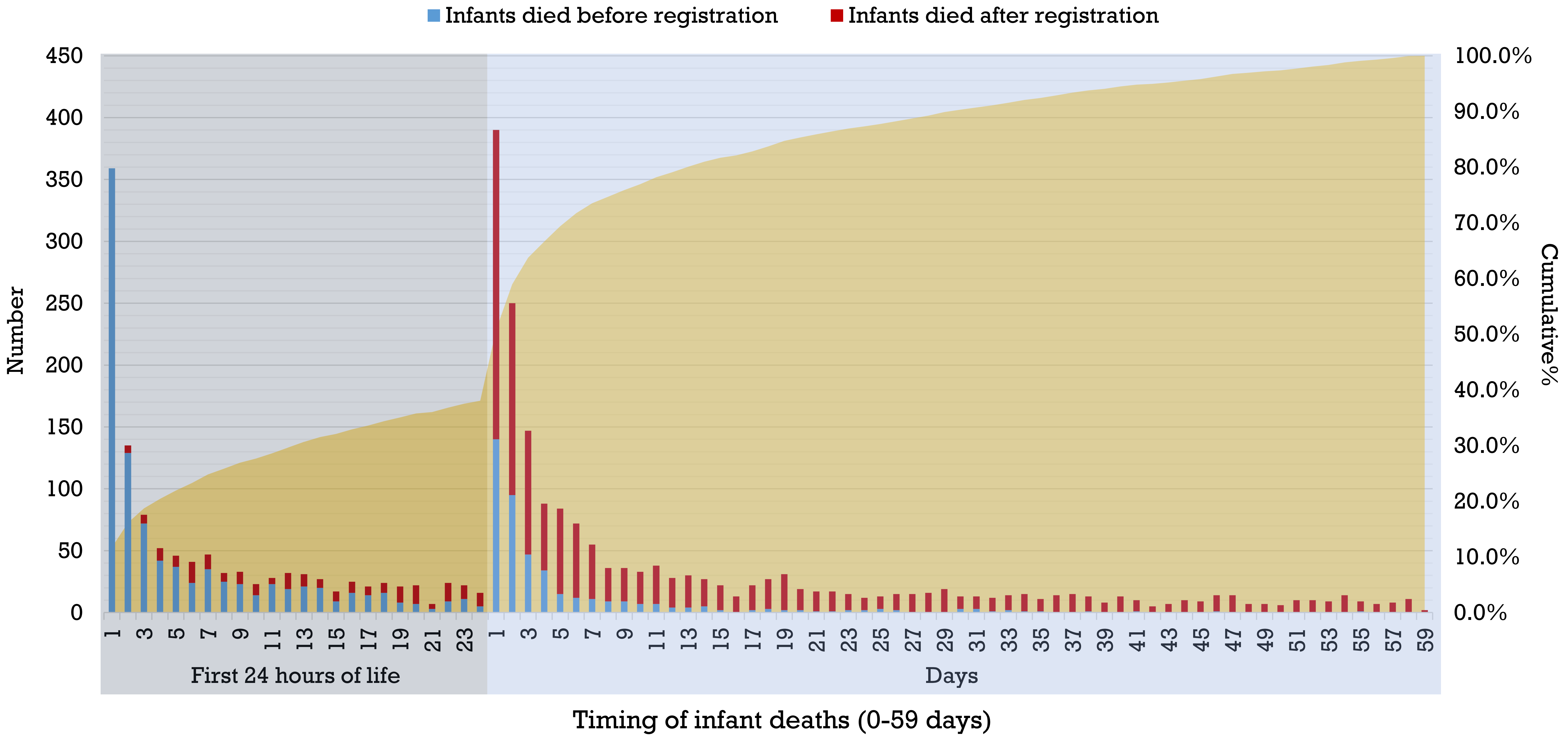
Infants died before registration
Infants died after registration
Number
Cumulative%
0
50
100
150
200
250
300
350
400
450
0.0%
10.0%
20.0%
30.0%
40.0%
50.0%
60.0%
70.0%
80.0%
90.0%
100.0%
First 24 hours of life
Days
Timing of infant deaths (0-59 days)

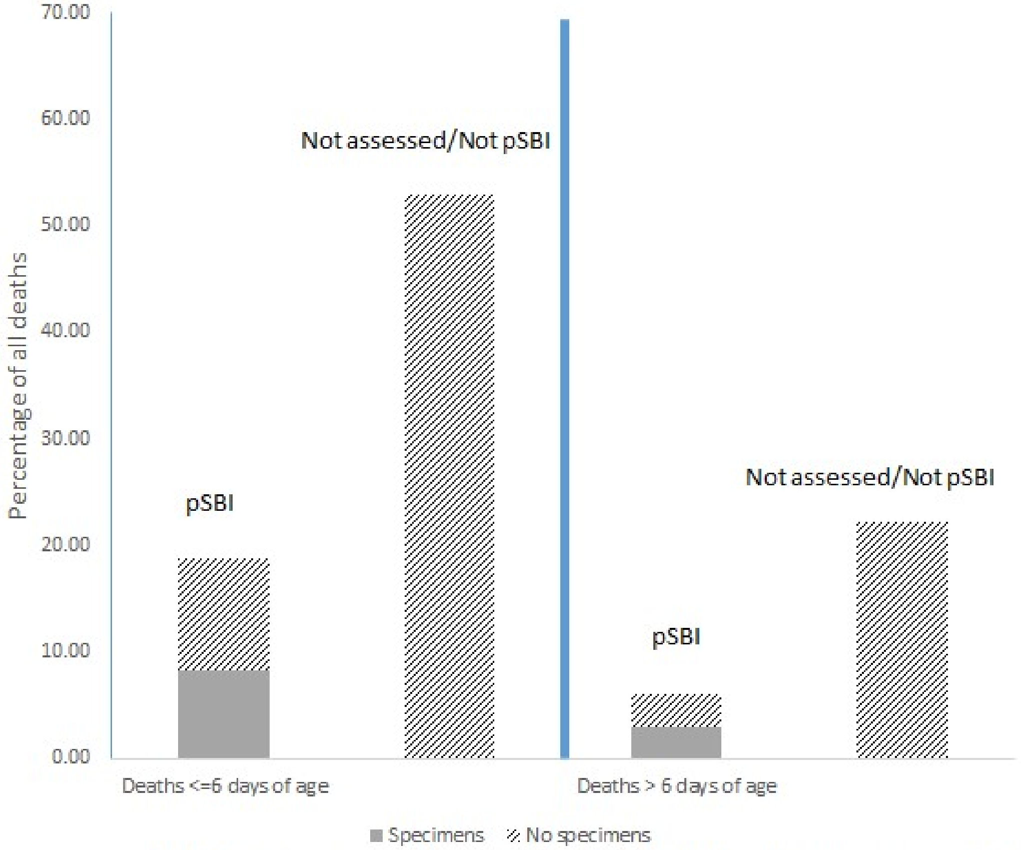

Infants died=3,061, Specimens collected=349, infant died within first 7 days of age=2195: pSBI with specimens=256; pSBI without specimens=323; not assessed=1,616, infants died after 7 days of age=866: pSBI with specimens=93; pSBI without specimens=96; not assessed=677

SM 14. Quality assurance plan for Taqman Array Cards (TAC) testing in Aetiology of Neonatal Infections in South Asia (ANISA) Study

In order to implement a quality assurance/quality control (QA/QC) program for molecular testing procedures at each ANISA site, the study coordination team requested shipment of 10% of all original specimens to the United States Centers for Disease Control and Prevention (CDC) for repeat extraction and TaqMan Array Card (TAC) testing. Each site shipped at least a 300 µL (0·3 mL) aliquot of every 10th whole blood specimen, and 400 µL (0·4 mL) aliquot of every 10th Nasopharyngeal/Oropharyngeal (NP/OP) swab and cerebrospinal fluid (CSF) specimen to CDC approximately quarterly each year through the duration of the study. Corresponding TAC run files for QA/QC specimens were also transferred to CDC electronically for comparison of results.

Among approximately 850 specimens retested at CDC, qualitative concordance of TAC results was greater than 80% among specimens with Ct values <30 for each assay on the study-specific TACs. Higher discordance among specimens with Ct values ≥30 was expected since target amplification occurred near the lower limit of detection for assays performed on TAC. Discordant results among these specimens likely occurred as a result of several factors, including exposure to additional freeze/thaw cycles, imperfect shipment conditions, extended storage times, and extraction and testing of a separate aliquot of original specimen. Repeat testing was repeated from original specimen, rather than residual extracted nucleic acid, in order to evaluate the entire specimen processing and testing procedure. Based on these factors, lower concordance was deemed acceptable within this subset of specimens.

Concordance, while one of the key criteria in identifying any issues in the laboratory, was just one aspect of the comprehensive data monitoring protocol implemented to ensure adherence to study protocols and quality of results. Comparison of results from testing performed at the study site and CDC enabled CDC to monitor performance and interpretation of controls, identify and troubleshoot issues with reagents, instruments, software, etc., identify potential laboratory contamination, track methodology changes, and/or identify training needs. If any issues were discovered after review of the criteria (Table 1), testing at a study lab were halted until the cause of the problem was resolved.

Table 1. Data monitoring parameters at study site level and laboratory level.

**Study site level parameters**
Total number of specimens of each type (NP/OP, blood, CSF) received
Total number of specimens of each type (NP/OP, blood, CSF) extracted
Average blood specimen extraction input volume and volume range[a]
Total number of specimens of each type tested on TAC
Number of positive results for each target for all blood and CSF specimens tested
Number of positive results for each target for all respiratory (NP/OP) specimens tested
Number of respiratory (NP/OP) specimens with no identified pathogen (*for cases only*)

Number of blood or CSF specimens with no identified pathogen (*for cases only*)
Mean Ct value(±standard deviation (SD)) and Ct range of RNaseP[b] reactions
Number of RNaseP reaction failures
Assays with 0 positive results to date
**Laboratory level parameters**
Total number of TACs of each type used
Number of Internal Positive Control (IPC)[c] reaction failures
Mean Ct value (±SD) and Ct range of IPC reactions
Mean Ct value (±SD) and Ct range of combined positive control for each target
Number of positive control failures; if yes, specify which target failed
Number of indeterminate results for each target
Positive results for NTC; if yes, specify target and Ct value
Threshold values for each target
Aberrant results (specify)
Assays with positive results for ≥ X% of specimens (variable by assay)

[a]When <300µL of whole blood was available due to limited volume obtained during blood draw, volume was adjusted up to 300 µL using neutral buffer solution. Actual volume of blood input was recorded for each specimen.

[b]RNaseP is a human-specific target used as a control for specimen collection, nucleic acid extraction, and real-time PCR performance.

[c]IPC served as a control for real-time PCR setup and performance.
